# Isolation and characterization of H9N2 influenza virus isolates from poultry respiratory disease outbreak

**DOI:** 10.1186/2193-1801-3-196

**Published:** 2014-04-18

**Authors:** Subhash J Jakhesara, Vaibhav D Bhatt, Namrata V Patel, Kantilal S Prajapati, Chaitanya G Joshi

**Affiliations:** Department of Animal Biotechnology, College of Veterinary Science & Animal Husbandry, Anand Agricultural University, Anand, Gujarat 388001 India; Department of Veterinary Pathology, College of Veterinary Science & Animal Husbandry, Anand Agricultural University, Anand, Gujarat 388001 India

**Keywords:** Avian influenza, Phylogeny, Poultry, Next generation sequencing, Virus isolation

## Abstract

The present study reports isolation and characterization of H9N2 virus responsible for disease characterized by symptoms including difficulty in respiration, head swelling, nasal discharge, reduced feed intake, cyanotic comb, reduced egg production and mortality. Virus isolation from allantoic fluid inoculated with tracheal aspirates and whole genome sequencing of two isolates were performed on an Ion-Torrent sequencer. Phylogenetic analysis revealed that the two H9N2 isolates are reassortant viruses showing a G1-like lineage for HA, NA and NP, a Hok/49/98-like lineage for PB1 and PA, PK/UDL-01/05-like lineage for PB2, IL/90658/00-like lineage for NS and an unknown lineage for M gene. Analyses of the HA cleavage site showed a sequence of (333PARSSR↓GL340) indicating that these isolates are of low pathogenicity. Isolate 2 has leucine at amino acid position 226, a substitution which is associated with mammalian adaptation of avian influenza virus. Isolate 1 has the S31N substitution in the M2 gene that has been associated with drug resistance as well as R57Q and C241Y mutations in the NP gene which are associated with human adaptation. The result reported here gives deep insight in to H9N2 viruses circulating in domestic poultry of India and supports the policy of active efforts to control and manage H9N2 infections in Indian poultry.

**Electronic supplementary material:**

The online version of this article (doi:10.1186/2193-1801-3-196) contains supplementary material, which is available to authorized users.

## Introduction

Influenza viruses are negative strand RNA viruses of the Orthomyxoviridae family that cause a variety of infections of high and low pathogenicity in hosts that include mammalian and avian species. Influenza viruses are divided in to types A, B and C. Type B and Type C cause mild infection only in human being and show limited genetic diversity as they are restricted to humans. In contrast type A viruses co-circulate in different host species and display considerable genetic diversity that is acquired through genetic reassortments and mutations. Over time a variety of influenza A subtypes have adapted to poultry hosts from wild birds and these can pose serious zoonotic risks to humans. Since the initiation of influenza surveillance in 2001 India has observed infections from various subtypes including H11N1 (Pawar et al. 2010), H4N6 (Pawar et al. 2012), H5N1 (Mishra et al. 2009), H9N2 (Tosh et al. 2008) in avian; H1N1, H3N2 (Chadha et al. 2012) H5N1 (ABY27653, unpublished) in humans and H3N8 (Virmani et al. 2010) in horse. H9N2 is enzootic in birds in India and causes infection with low pathogenicity.

H9N2 infection in domestic poultry was reported during 2003–04 in poultry farms with history of reduced egg production and respiratory illness in Punjab, Haryana, Uttar Pradesh, Gujarat and Orissa states of India (Nagarajan et al. 2009). Similar cases of respiratory illness were reported from layer poultry farms in the Sarsa village of Gujarat, India. Clinical disease signs, such as difficulty in breathing, head swelling, nasal discharge, reduced feed intake and cyanotic comb, were recorded and affected flocks showed illness for 3–4 weeks with a drop in egg production of up to 40%. Approximately, 2–3% and 10–30% of mortality was observed in affected grower and layer flocks, respectively. Postmortem examination revealed fibrino-caseative material in the infra-orbital sinus, congested tracheal mucosa with caseous material, oviduct wall edema, fibrin plaques on the ovary and caseous plugs in the tracheal bifurcation.

The genetic, antigenic and evolutionary characterization of H9N2 viruses circulating in India is not well documented. Here, to explore the genetic and phylogenetic nature of H9N2 viruses in India, we have isolated H9N2 viruses from clinically affected birds and used whole genome sequencing to analyze two independent isolates at the molecular level. In particular we have examined these isolates for molecular signatures indicative of potential adaptation of viruses to mammalian host species.

## Materials and methods

### Virus isolation

Tracheal aspirates and swabs were collected from dead chickens during pathological examination. The collected clinical specimens were centrifuged for 10 minutes at 8, 000 × g, and after removing bacterial contaminants by millipore membrane filters (0.22 μm), the supernatant was incubated in the allantoic cavities of 10-day-old specific pathogen free (SPF) chicken eggs at 37°C for 72 h for viral isolation; finally the allantoic fluids were harvested and stored at −80°C.

### Viral RNA extraction and sequencing

Isolation of viral RNA was done from virus containing allantoic fluid using a QIAmp viral RNA mini kit (Qiagen, Germany). The quality, quantity and integrity of total RNA was evaluated using a NanoDrop1000 spectrophotometer (Thermo Fisher Scientific, DE) and a Bioanalyzer 2100 (Agilent Technologies, CA). Total RNA was subjected to emulsion PCR after library preparation and DNA-positive beads from emulsion PCR were recovered, enriched and subjected to Ion semiconductor sequencing using a 316 chip on an Ion-Torrent sequencer following manufacturer’s protocol.

### Quality filtering and mapping of reads

Available reads were subjected to quality filtering by PrinseQ (Schmieder and Edwards 2011) based on the criteria of a minimum read length 40 and mean base quality for the entire read of 25. Quality filtered reads were assembled against the genome sequence of H9N2 strain A/Quail/Hong_Kong/G1/97 using CLC genomics workbench v4.7.1 (http://www.clcbio.com/products/clc-genomics-workbench/).

### Genetic and phylogenetic analysis

Sequence analysis was conducted using Mega 5.2 (Tamura et al. 2011). Nucleotide sequence alignments were constructed for each of the eight genomic segments using the MUSCLE algorithm in Mega 5.2. The H9N2 isolates in the current study, the H9N2 virus strains isolated from India, all available Asian strains with full genome sequences from avian and humans and three different H9N2 virus lineages specified by the National Center for Biotechnology Information (NCBI) influenza database were included in the analysis. All sequence data were downloaded from the NCBI influenza database. The neighbour-joining method with Kimura two-parameter distances was used to construct the phylogenetic trees using Mega 5.2 (Kimura 1980). The reliability of internal branches was assessed by the p-distance substitution model using 1000 bootstrap replications.

### Deduced amino acid sequence analysis

Consensus sequences of each segment were subjected to blastx against the NCBI flu database. Regions of amino acid sequences showing maximum match against flu protein sequences were considered for further analysis. Amino acid sequences from each segment were subjected to multiple alignments using MUSCLE. To determine variations at the sites of viral attachment, we used a web based application, ScanProsite (de Castro et al. 2006) to compare and identify N-glycosylation sites and determined differences with other strains.

## Results

### Sequencing of influenza virus isolates

A total of 14,914 and 314,197 reads were obtained respectively for isolate1 and isolate 2. After stringent quality filtering and duplicate removing 4,457 (29.88%) and 137,675 (43.82%) sequences were assembled against genome sequence of H9N2 strain A/Quail/Hong Kong/G1/97. Alignments were manually observed for homopolymer and indel verification and curated consensus sequences were produced for each segment of both the isolates.

### Phylogenetic analysis

Eight gene segments of the 2 isolates were phylogenetically analyzed against full length sequences of 199 Asian viruses as well as all available Indian H9N2 viral sequences (Additional file 1). Lineages for each of segment were assigned according to the previous study on comprehensive phylogenetic analysis of H9N2 viruses isolated from 1966 to 2009 (Dong et al. 2011). The phylogenetic tree of the HA gene (segment 4) revealed that both isolates were grouped together with other Indian strains (Additional file 1: Figure S1A). The phylogenetic tree of HA genes revealed that the G1 like lineage (represented by A/Quail/Hong Kong/G1/97) is the lineage under which all the Indian viruses can be grouped. A Similar profile was obtained for the NA gene (segment 6) and all Indian strains together with two isolates can be grouped under G1 like lineage (Additional file 1: Figure S1B). Similar to HA and NA, the phylogenetic tree of the NP gene (segment 5) revealed that all Indian strains could be grouped under G1 like lineage (Additional file 1: Figure S1F). The PB2 gene (segment 1) of Pakistan/UDL-01/2005 like lineage was predominant in the Indian strains (Additional file 1: Figure S1C). Phylogenetic tree for PB1 (segment 2; Additional file 1: Figure S1D) and PA (segment 3; Additional file 1: Figure S1E) genes revealed that all the Indian H9N2 strains together with two isolates from this study could be grouped under the Hok/49/98 like lineage for both the genes (represented by A/duck/Hokkaido/49/98). For NS gene (segment 8; Additional file 1: Figure S1H), Indian strains together with two isolates clustered under IL/90658/00 like lineage (represented by A/ck/Israel/90658/2000). Interestingly for MP gene (segment 7; Additional file 1: Figure S1G) no known lineage can be assigned and all Indian strains examined, along with H9N2 strains from Pakistan and Israel, formed a distinct cluster.

### Molecular analysis of amino acid sequence

The deduced amino acid sequences of all genes were compared with representative Indian strains containing different HA and NA serotypes. It has been shown that the presence of multiple basic amino acids at the cleavage position increases cleavage of HA in to HA1 and HA2 and may result in increased pathogenicity (Kawaoka and Webster 1988). Deduced amino acid sequences at the cleavage site of both the isolates lacked multiple basic amino acids; comparison to other Indian H9N2 strains revealed a similar RSSR motif with exception of some strains in which the fourth amino acid upstream from the cleavage site was mutated to Lysine (K)/Valine (V) (Table 1). It needs to be studied whether this change in cleavage site has any effect on pathogenicity. It is considered that glycosylation sites affect the receptor binding capacity of HA (Ohuchi et al. 1997). Analysis of HA proteins of G1 lineage strain has revealed 8 potential N-glycosylation sites at 29, 105, 141, 206, 218, 298, 305, and 492 whilst the isolates identified in this study lack two potential glycosylation sites present at 206 and 218. It has been suggested that glycosylation patterns plays an important role in the adaptation of avian influenza virus to poultry (Matrosovich et al. 1999) hence it is possible that these changes in sites may contribute to selective avian adaptation.

**Table 1:** **Comparison of amino acid residues at specific positions in the HA and NA gene**

	HA					NA					
Strain	Receptor binding site				Cleavage site	Stalk deletion		HB site			
	189	190	226	227	333–340	38–39	62–65	366–373	402	403	432
Isolate1	T	A	L	I	PARSSR↓GL	No	No	IKKDSRAG	N	W	R
Isolate2	T	A	Q	I	PARSSR↓GL	No	No	IKKDSRAG	N	W	Q
A/chicken/Chandigarh/2048/2003	T	A	L	I	PARSSR↓GL	No	No	IKKDSRAG	N	W	NA
A/chicken/Haryana/2424/2004	T	A	L	I	PARSSR↓GL	No	No	IKKDSRAG	N	W	NA
A/chicken/India/IVRI-0004/2010	T	A	L	I	PAKSSR↓GL	No	No	IKRDSRAG	N	W	Q
A/chicken/India/WB-NIV1057231/2010	T	V	L	I	PARSSR↓GL	No	No	IKEDSRAG	N	W	Q
A/chicken/Orissa/2317/2004	T	A	L	I	PARSSR↓GL	No	No	IKKDSRAG	N	W	NA
A/chicken/Tripura/105131/2008	T	A	L	I	PAKSSR↓GL	No	No	INKDSRAG	N	W	R
A/chicken/Uchal/8293/2006	T	A	L	I	PARSSR↓GL	No	No	IRKDSREG	N	L	Q
A/watercoot/Haryana/5844/2005	T	A	L	I	PARSSR↓GL	No	No	IKKDSRAG	N	W	Q
A/Quail/Hong Kong/G1/97 H9N2	T	E	L	Q	PARSSR↓GL	Yes	No	IKKDSRSG	I	R	Q
A/duck/Hong Kong/Y280/97H9N2	T	T	L	Q	PARSSR↓GL	No	Yes	IKEDSRSG	N	W	Q
A/turkey/Wisconsin/1/1966H9N2	T	E	Q	Q	PAVSSR↓GL	No	No	ISKDSRSG	N	W	Q

Receptor binding site in HA protein was also analyzed with respect to amino acid changes at positions 189,190,226 and 227 (H3 numbering, 197,198,234 and 235 for H9 numbering). Both the current isolates together with all Indian H9N2 strains (excluding A/ck/India/WB-NIV1057231/2010-Valine) have alanine at position 190, compared to the three characterized H9N2 lineage strains (G1, Y280, WI1) which contain glutamic acid or threonine. Additionally, all the Indian strains (excluding A/duck/India/31g/86-Q) and both the current isolates have isoleucine (I) at position 227 (H3 numbering, 235 for H9 numbering) in contrast to glutamine (Q) in all three lineage strains (Table 1). Most importantly the substitution Q226L was observed in isolate 2 in present study, which is associated with mammalian adaptation of avian influenza virus.

The deduced amino acid sequence of the NA protein was compared with that of Indian isolates and lineage defining strains. None of the current isolates or any of the other Indian strains possesses NA stalk deletions at positions 38–39 and 63–65 in the G1 and Y280 lineages respectively. Sequence analysis of the sialic acid-binding haemabsorbing (HB) site located on the surface of the NA molecule at positions 366–373, 400, 403 and 432 (Kobasa et al. 2001) is well conserved with some exceptions. A common substitution resulting in change of serine to alanine or glutamic acid was observed at position 372 amongst the isolates and Indian strains, respectively. Isolate1 along with only one Indian strain (A/ck/Tripura/105131/08) also revealed a unique substitution from glutamine to arginine at position 432 when compared with strains of G1 and Y280 lineage. Comparison of conserved potential N-glycosylation sites in the NA glycoprotein showed that the lineage strains contain seven sites at positions 61, 69, 86, 146, 200, 234 and 402. The isolates sequenced in this study as well as most of the Indian isolates revealed an additional site at asparagine (N) 44 due to substitution of P45S.

Several amino acid changes present in other proteins of influenza virus are known to play roles as molecular determinants for host range and virulence. Several such determinants have been described in various studies (Finkelstein et al. 2007; Shaw et al. 2002; Chen et al. 2006; Manzoor et al. 2009; Naffakh et al. 2008; Katz et al. 2000). The isolates sequenced in this study were also analyzed for these signatures in different genes (PB1, PB2, PA, NP, MA and M2) and results are described in Table 2. Substitutions of 2, 1, 2, 3, 1, 3 and 1 residues from avian associated H9 viruses to human associated H9 viruses (or other amino acids) were found in genes PB2, PB1, PA, NP, M1, M2 and NS1 respectively in current isolates. NS2 protein did not revealed any substitution frequently observed in human associated influenza viruses.

**Table 2:** **Analysis of mutation at amino acid positions important for host range pathogenicity and virulence**

Protein	aa position	Deduced amino acid		Amino acid in isolates from this study	Reference
		Human	Avian		
PB2	44	S	A	A	(Chen et al. 2006; Finkelstein et al. 2007; Shaw et al. 2002)
	64	T	M	M	(Finkelstein et al. 2007)
	81	M	T	T	(Shaw et al. 2002)
	199	S	A	A	(Chen et al. 2006; Shaw et al. 2002; Finkelstein et al. 2007)
	256	G	D	D	(Manzoor et al. 2009)
	271	A	T	T	(Chen et al. 2006; Shaw et al. 2002)
	333	I	T	T	(Naffakh et al. 2008)
	355	Q	K	**R** ^**a**^	(Katz et al. 2000)
	475	M	L	L	(Chen et al. 2006)
	482	R	K	K	(Naffakh et al. 2008)
	588	I	A	**V**	(Chen et al. 2006; Shaw et al. 2002; Finkelstein et al. 2007)
	613	T	V	V	(Chen et al. 2006; Shaw et al. 2002)
	627	K	E	E	(Chen et al. 2006; Naffakh et al. 2008)
	661	T	A	A	(Shaw et al. 2002)
	674	T	A	A	(Chen et al. 2006; Shaw et al. 2002; Finkelstein et al. 2007)
	701	N	D	D	(Naffakh et al. 2008)
	702	R	K	K	(Shaw et al. 2002)
	714	R	S	S	(Naffakh et al. 2008)
PB1	13	P	L	**P**	(Naffakh et al. 2008)
	327	K	R	R	(Chen et al. 2006)
	336	1	V	V	(Chen et al. 2006)
	538	G	D	D	(Naffakh et al. 2008)
	578	Q	K	K	(Naffakh et al. 2008)
	678	N	S	S	(Naffakh et al. 2008)
PA	28	L	P	P	(Chen et al. 2006; Shaw et al. 2002)
	55	N	D	D	(Chen et al. 2006; Shaw et al. 2002)
	57	Q	R	**Q**/R	(Chen et al. 2006; Finkelstein et al. 2007)
	65	L/Y	S	S	(Naffakh et al. 2008; Shaw et al. 2002)
	100	A	V	V	(Shaw et al. 2002)
	133	G	E	E	(Naffakh et al. 2008)
	241	Y	C	**Y**/C	(Shaw et al. 2002)
	225	C	S	S	(Chen et al. 2006)
	268	I	L	L	(Chen et al. 2006)
NP	16	D	G	G	(Chen et al. 2006)
	31	K	R	R	(Shaw et al. 2002)
	33	I	V	V	(Chen et al. 2006; Shaw et al. 2002)
	34	N	D	**G**	(Naffakh et al. 2008)
	61	L	I	I	(Chen et al. 2006; Shaw et al. 2002)
	100	V	R	R	(Chen et al. 2006; Shaw et al. 2002)
	109	V	I	I	(Chen et al. 2006)
	127	D	E	E	(Shaw et al. 2002)
	136	M	L	**I**	(Shaw et al. 2002)
	214	K	R	R	(Chen et al. 2006; Shaw et al. 2002)
	283	P	L	L	(Chen et al. 2006; Shaw et al. 2002)
	319	K	N	N	(Naffakh et al. 2008)
	293	K	R	R	(Chen et al. 2006; Shaw et al. 2002)
	305	K	R	R	(Chen et al. 2006)
	313	Y	F	F	(Chen et al. 2006; Shaw et al. 2002)
	357	K	Q	Q	(Chen et al. 2006; Finkelstein et al. 2007)
	372	D	E	**D**	(Chen et al. 2006)
	375	G/E	D	D	(Shaw et al. 2002)
	422	K	R	R	(Chen et al. 2006)
	442	A	T	T	(Chen et al. 2006)
	455	E	D	D	(Chen et al. 2006)
	480	N	D	D	(Naffakh et al. 2008)
M1	15	I	V	**I**	(Katz et al. 2000)
	115	I	V	V	(Chen et al. 2006)
	121	A	T	T	(Chen et al. 2006)
	137	A	T	T	(Shaw et al. 2002)
M2	11	I	T	T	(Chen et al. 2006)
	16	G/D	E	**G**	(Shaw et al. 2002)
	20	N	S	S	(Chen et al. 2006; Shaw et al. 2002)
	28	I/V	I	**F**	(Shaw et al. 2002)
	55	F	L	**F**	(Shaw et al. 2002)
	57	H	Y	Y	(Chen et al. 2006)
	78	K	Q	Q	(Shaw et al. 2002)
	86	A	V	V	(Chen et al. 2006)
NS1	215	T	P	P	(Finkelstein et al. 2007)
	227	R/K	E	**K**	(Chen et al. 2006; Finkelstein et al. 2007)
NS2	70	G	S	S	(Chen et al. 2006)

^a^Amino acids in boldface denotes the change from nucleotide commonly observed in avian associated H9 viruses.

## Discussion

Avian influenza is a serious threat to the poultry industry’s economy and continuous surveillance and vaccination are two main factors in control of disease. Since 2006, India has experienced three outbreaks that caused severe economic losses (Chakrabarti et al. 2009). As a response to these outbreaks, surveillance of avian influenza in India has become a priority and several reports of avian influenza surveillance have been published (Pawar et al. 2012; Rao 1987). Avian influenza surveillance has confirmed the H9N2 strain as predominant along with H4N6, causing low pathogenic avian influenza in wet poultry markets and backyard poultry in West Bengal. Because influenza A viruses continue to evolve, the identification and characterization of local strains circulating at regional level is of paramount importance to keep a constant watch on influenza enzootics. The genetic nature of H9N2 viruses showing potential human receptor binding specificity and increasing reports of H9N2 viruses in India, along with newly emerging reassortant viruses, puts H9N2 in the list of viruses posing potential threat to humans.

This study represents a comprehensive, in-depth analysis of H9N2 viruses isolated from Gujarat, India. We could show that both isolates in this study are reassortant in nature; i.e. they contain more than one lineage for the eight segments present in their genome. Lineage assignment revealed that H9N2 viruses circulating in Gujarat comprise genetically similar segments from viral strains of other Asian countries such as China, Japan, Israel and Pakistan. This may be attributed to typical routes taken by migratory wild birds migrating from east-Asia through India, giving opportunities for viruses to exchange segments during co-circulation (Pawar et al. 2012). Phylogenetic analysis revealed an unknown lineage for the MP gene which was further separated in to two sub lineages consisting of strains reported before and after 2008 with the exception of two strains A/ck/India/IVRI-004/2010 and A/ck/India/IVRI-0011/2011 which were grouped with strains isolated before 2008. To check the homology of the MP gene of our isolates to strains other than H9N2 we performed blast and blastx of segment 7 nucleotide sequence. We found 96% and 99% of homology to a H7N3 strain from Pakistan (A/chicken/Karachi/NARC-100/2004) at nucleotide and protein level, respectively. Indeed, it has been found from several studies of H9N2 strains from Pakistan (Iqbal et al. 2009) and China (Guan et al. 2000), that reassortant H9N2 strain contains segments from highly pathogenic H5N1 or H7N3 strains. As LPAI H9N2 is widely prevalent in domestic poultry, it is possible that novel reassortant virus with high pathogenicity may arise and the virus may infect humans. This warrants influenza surveillance of migratory birds coming to India for validation.

The host range and adaptation to different host is correlated with amino acids present within and around the HA receptor binding site (RBS) which can confer different receptor binding specificity (Weis et al. 1988). In particular the amino acids glutamine (Q) or leucine (L) at position 226 (H3 numbering, 234H9 numbering) plays a key role in avian and human virus-like receptor specificity respectively. The presence of leucine (L) at position 226 is associated with mammalian adaptation avian influenza virus. In our study except for position 226, no additional changes were observed in the HA RBS that could enable avian-human transmission. Similar type of observations were also reported for HA gene of H9N2 viruses prevalent in Israel after detailed molecular analysis and it was found that H9N2 viruses circulating in the Israel are predominantly of G1 lineage and harbors Q226L substitution in HA gene (Davidson et al. 2013,2014). The Q226L substitution seen in RBS of H9N2 avian isolates is important as it confers human virus like receptor specificity and binds more strongly with 2-6-linked sialic acid receptors as seen in human H3N2 viruses (Matrosovich et al. 2001) in contrast to viruses lacking this substitution which binds more strongly with 2-3 linked sialic acid receptors. To check when this substitution was first introduced in Indian isolates we compared amino acid sequences of the HA gene and we found that H9N2 virus was isolated for the first time in India from the duck in 1986 which did not contain this substitution. Thereafter again it was detected in 2003 from Chandigarh and Haryana state of India and we found that these viruses which introduced after 2003 contained Q226L substitution. Our analysis found that since 2003 Q226L substitution is prevailing in the H9N2 viruses predominantly of G1 like lineage isolated from the India. This report shows need of continuous surveillance and reporting of the molecular changes occurring in the avian H9N2 viruses which can confer human virus like receptor specificity.

Taking account of amino acid changes in other internal genes of the isolates sequenced in this study, 2, 1, 2, 3, 1, 3 and 1 substitutions were found in the PB2, PB1, PA, NP, M1, M2 and NS1 genes. The PB2 gene in both isolates revealed K355R and A588V substitutions which are not associated with human associated H9 viruses. Additionally, both viruses have a L13P substitution frequently observed in human associated H9 viruses, but it is important to note that this change is commonly observed in H9N2 strains. The NP gene revealed D34G, L136I and E372D substitutions frequently seen in human associated H9 viruses. Considering the PA gene, isolate1 revealed R57Q and C241Y substitutions, which are associated with human adaptation. However, isolate 2 has retained avian associated amino acids at these positions. Whether these changes in PB2, PB1, PA and NP genes will cause increased pathogenicity and virulence in chickens or have emerged as a result of adaptation to hosts other than avian remains to be investigated, nonetheless substitutions at these positions are considered to influence replication efficiency and may act as hallmarks of host-specific adaptation. The M1 protein revealed V15I substitution in both isolates which is common in all the H9N2 lineages. The M2 gene has been extensively studied for drug resistance several mutations are known to confer drug resistance; L26F, V27A, A30T, S31N, G34E, L38F (Schnell and Chou 2008). Isolate 1 from this study has the S31N substitution, which has also been identified in H9N2 viruses from Iran, UAE and Qatar (Fusaro et al. 2011). Analyses of additional host range signatures for M2 gene in both isolates revealed I28F, E16G and L55F substitutions commonly observed in human associated H9 viruses. These changes in M1 and M2 proteins are likely to confer phenotypes of increased replication efficiency and amantadine resistance. Our analysis of the NS1 protein revealed E227K substitution in both the isolates, a substitution that confers increased pathogenicity and virulence in mice (Jackson et al. 2008). This lies within the C-terminal 227KSEV230 PDZ binding motif that is implicated in virus inhibition of apoptosis and overcoming host interferon responses (Thomas et al. 2011). E227K substitution is observed in some of the H5N1 viruses isolated from Indonesia and Saudi Arabia (Obenauer et al. 2006; Monne et al. 2008). However, it remains to be investigated whether E227K substitution results in increased virulence of H9N2 viruses in avian species. It is important to note here that we assessed these substitutions probably associated with host range and pathogenicity for H9N2 viruses identified in present study only, whether these substitutions are prevailing in other zoonotic influenza subtypes and contributes towards variation in pathogenicity needs further study and validation.

## Conclusion

This study represents comprehensive genetic and phylogenetic characterization of H9N2 viruses recently isolated from chickens in Gujarat, India. Phylogenetic analyses suggest that there is continuous evolution of H9N2 viruses with development of reassortant virus showing intra and inter subtype exchange of segments. A probable role for migratory birds in development of reassortant viruses could be validated by surveillance of avian influenza viruses in migratory birds and large scale sequencing efforts to characterize H9N2 in India. Further, the strains identified in this study harbor some substitutions which are frequently observed in human associated H9 viruses which need to be further studied by animal models for their effect on pathogenicity. The results reported herein gives deep insight in to H9N2 viruses circulating in domestic poultry of India, however there is a need for further characterization by sequencing a greater number of isolates. This study supports policies for the active control and management of H9N2 infections in Indian poultry markets to reduce the risk of transmission to human.

## Electronic supplementary material

Additional file 1: **Phylogenetic trees of all eight genes of H9N2 avian influenza.** Phylogenetic relationships for HA (A), NA (B), PB2 (C), PB1 (D), PA (E), NP (F), MP (G) and NS (H) genes of the 223 analyzed H9N2 influenza viruses along with 2 isolates from present study. Genome sequences of 223 H9N2 viruses from Asian strains and reference lineage strains were selected from the NCBI Influenza Virus Resource Database. Neighbor-joining trees were constructed using MEGA and bootstrap values are shown for the key nodes. Clade labelled using different color indicate viruses clustered as Indian isolates together with isolates form this study. Representative viruses for each lineage and isolates form this study are presented in blue color. (PDF 186 KB)
