# Supplementary material for: Isolation and characterization of H9N2 influenza virus isolates from poultry respiratory disease outbreak
Source: Springerplus. 2014 Apr 18;3:196. doi: 10.1186/2193-1801-3-196 (PMC4004788; doi:10.1186/2193-1801-3-196)
Supplement: Supplementary file 1 — Additional file 1: Phylogenetic trees of all eight genes of H9N2 avian influenza. Phylogenetic relationships for HA (A), NA (B), PB2 (C), PB1 (D), PA (E), NP (F), MP (G) and NS (H) genes of the 223 analyzed H9N2 influenza viruses along with 2 isolates from present study. Genome sequences of 223 H9N2 viruses from Asian strains and reference lineage strains were selected from the NCBI Influenza Virus Resource Database. Neighbor-joining trees were constructed using MEGA and bootstrap values are shown for the key nodes. Clade labelled using different color indicate viruses clustered as Indian isolates together with isolates form this study. Representative viruses for each lineage and isolates form this study are presented in blue color. (PDF 186 KB) [file 40064_2014_914_MOESM1_ESM.pdf]

Title: Isolation and characterization of H9N2 influenza virus isolates from poultry respiratory disease outbreak

Journal name: SpringerPlus

S. J. Jakhesara [a,#], V. D. Bhatt [a], N. V. Patel [a], K. S. Prajapati [b], C.G. Joshi [a]

[a] *Department of Animal Biotechnology, College of Veterinary Science & Animal Husbandry, Anand Agricultural University, Anand, Gujarat, India.Pin: 388001*
[b] *Department of Veterinary Pathology, College of Veterinary Science & Animal Husbandry, Anand Agricultural University, Anand, Gujarat, India.Pin: 388001*

# corresponding author. Tel.: +91-02692-261201.

E-mail address: drsubhash81@gmail.com (S. J. Jakhesara)

Additional file 1- Phylogenetic trees of all eight genes of H9N2 avian influenza
Phylogenetic relationships for HA (A), NA (B), PB2 (C), PB1 (D), PA (E), NP (F), MP (G) and NS (H) genes of the 223 analyzed H9N2 influenza viruses along with 2 isolates from present study. Genome sequences of 223 H9N2 viruses from Asian strains and reference lineage strains were selected from the NCBI Influenza Virus Resource Database. Neighbor-joining trees were constructed using MEGA and bootstrap values are shown for the key nodes. Clade labelled using different color indicate viruses clustered as Indian isolates together with isolates form this study. Representative viruses for each lineage and isolates form this study are presented in blue color.

Fig S1A HA

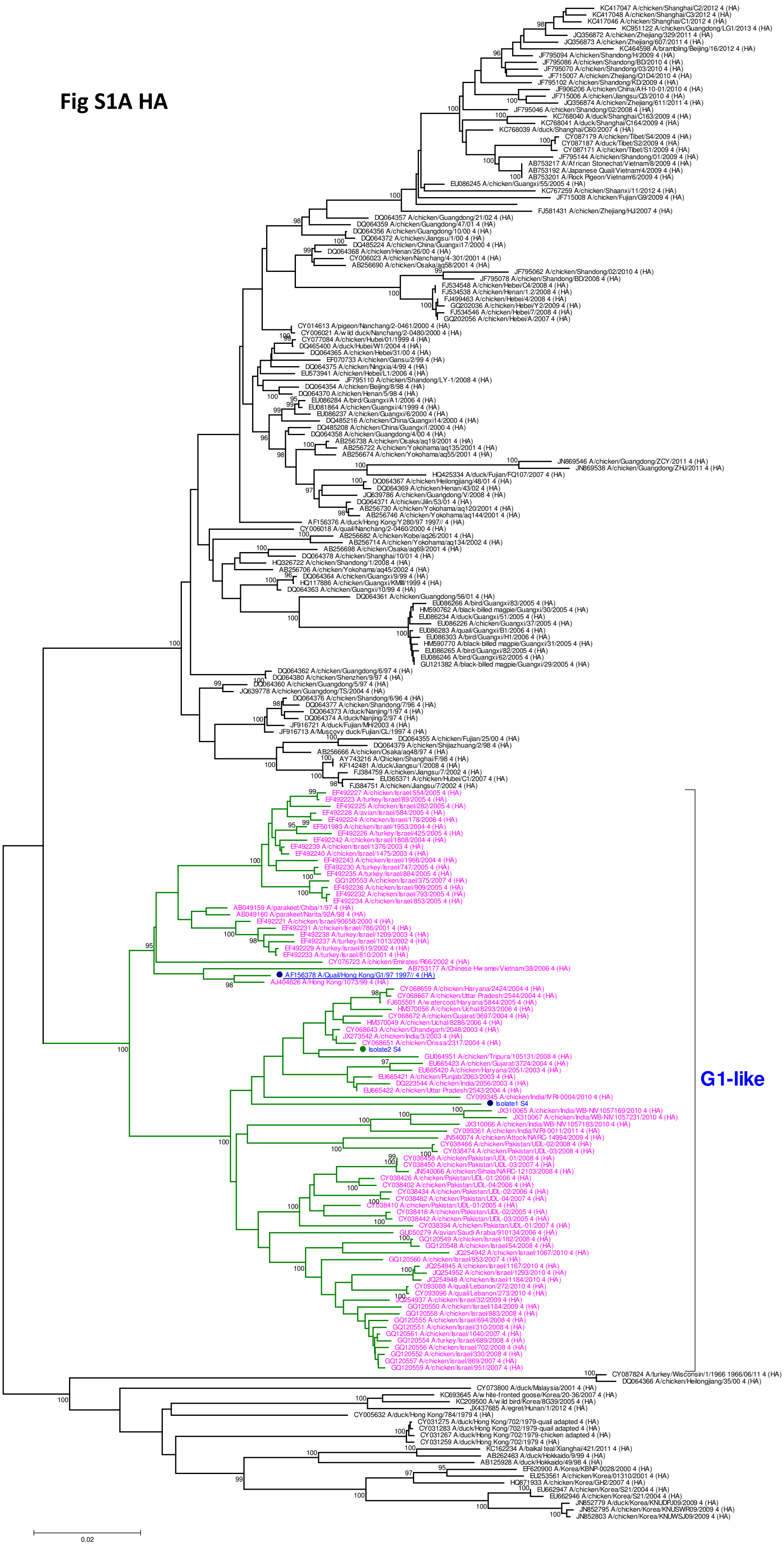

Fig S1B NA

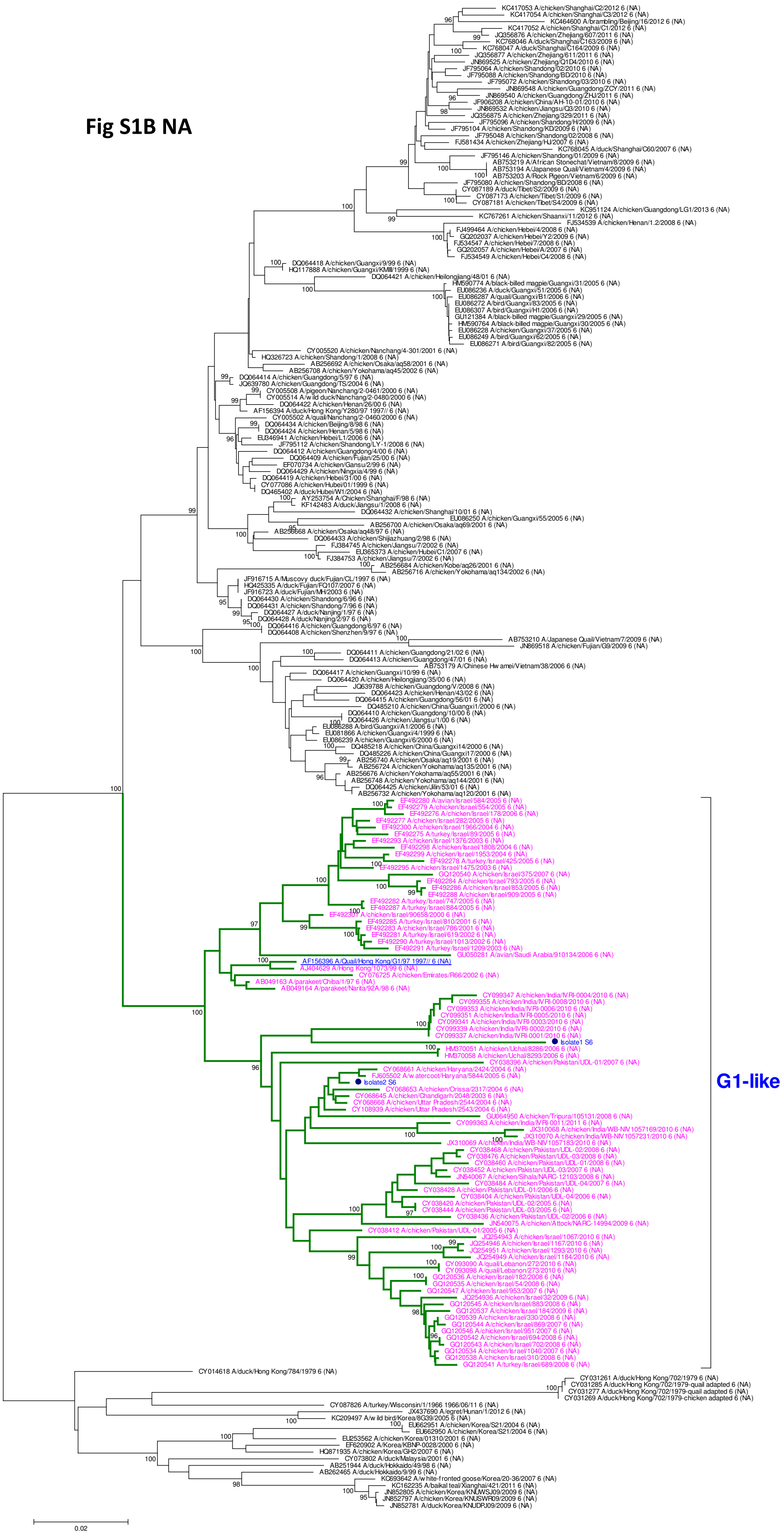

Fig S1C PB2

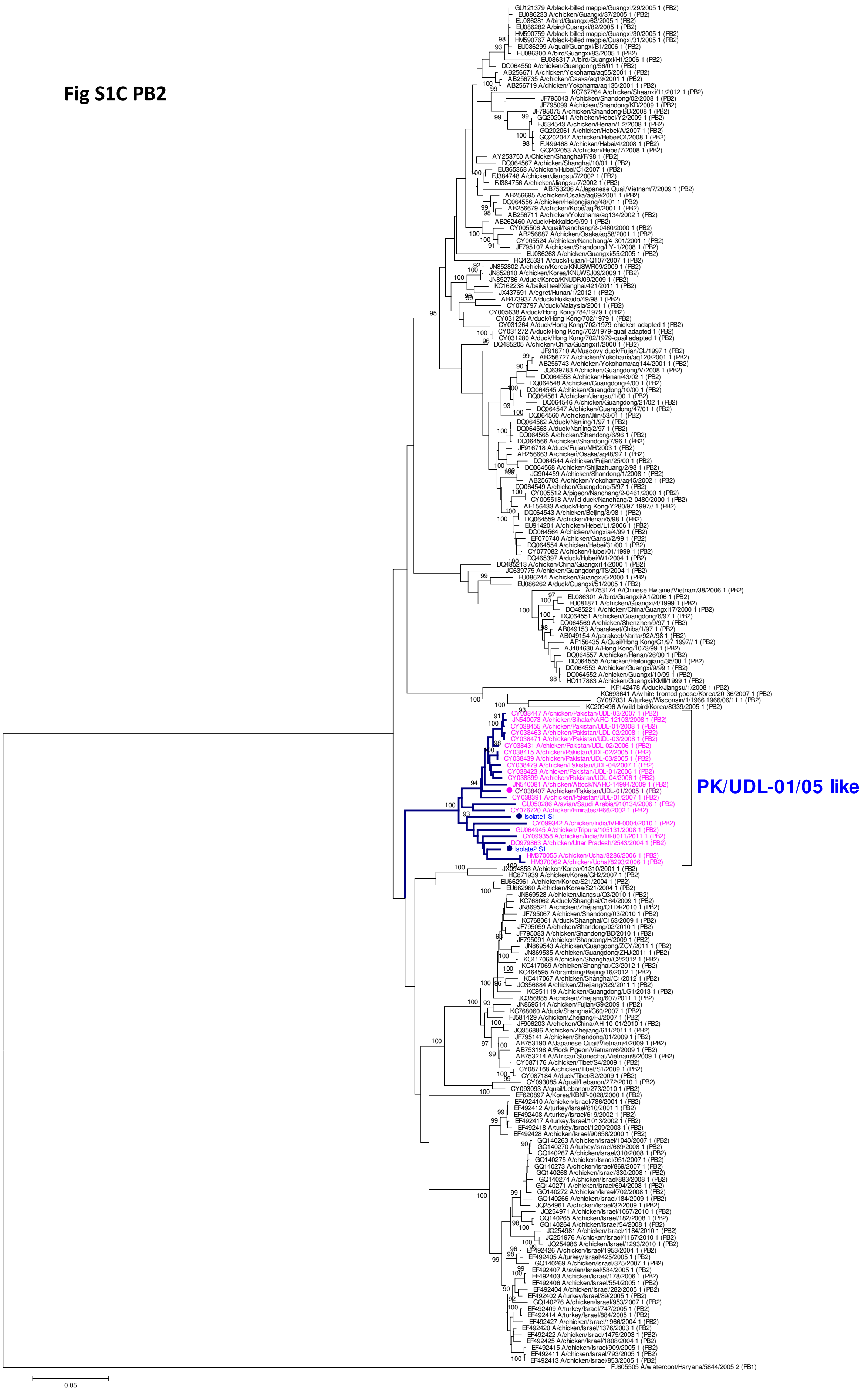

Fig S1D PB1

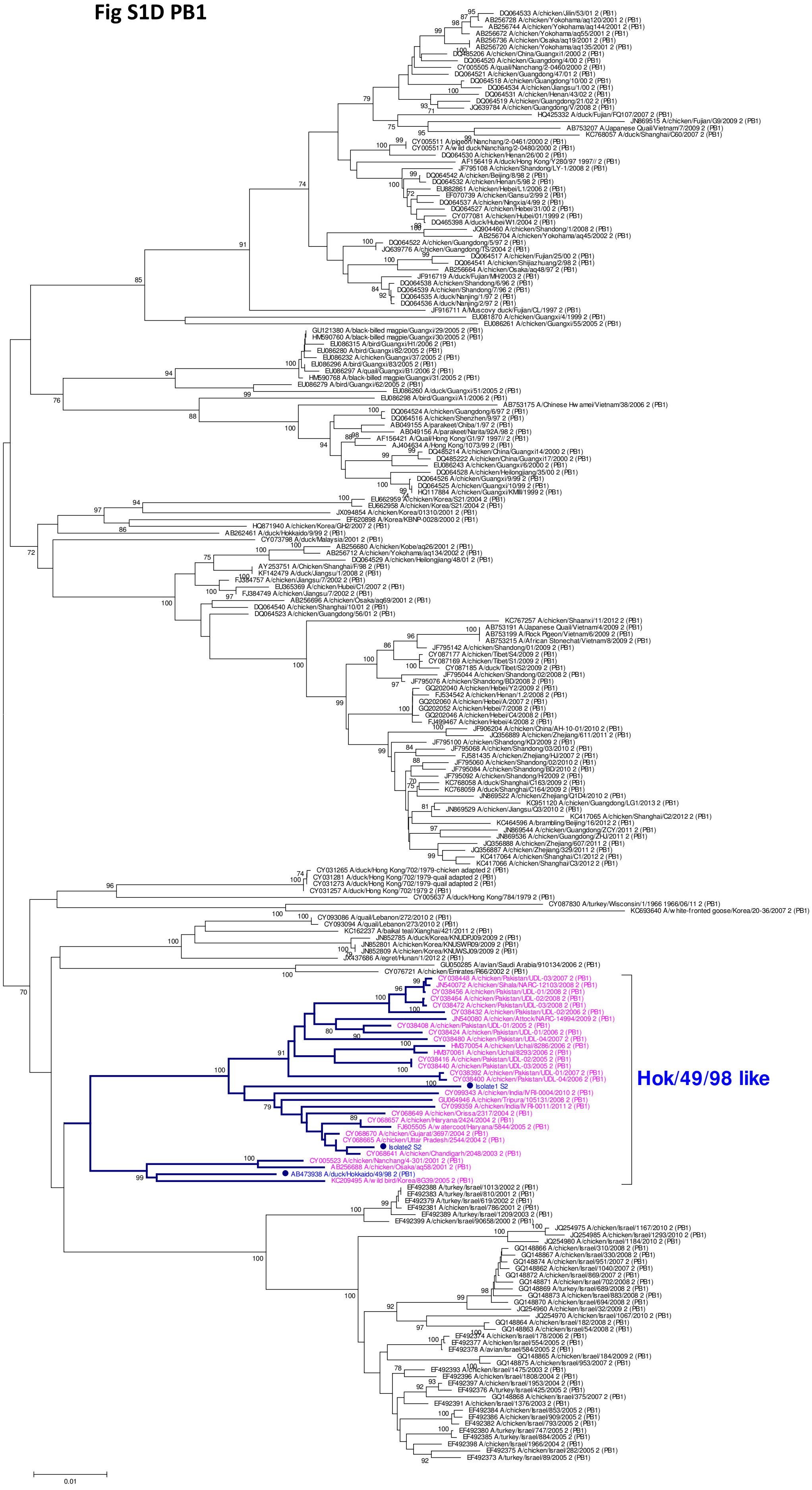

Hok/49/98 like
Isolate1 S2
Isolate2 S2
AB473938 A/duck/Hokkaido/49/98 2 (PB1)
0.01

Fig S1E PA

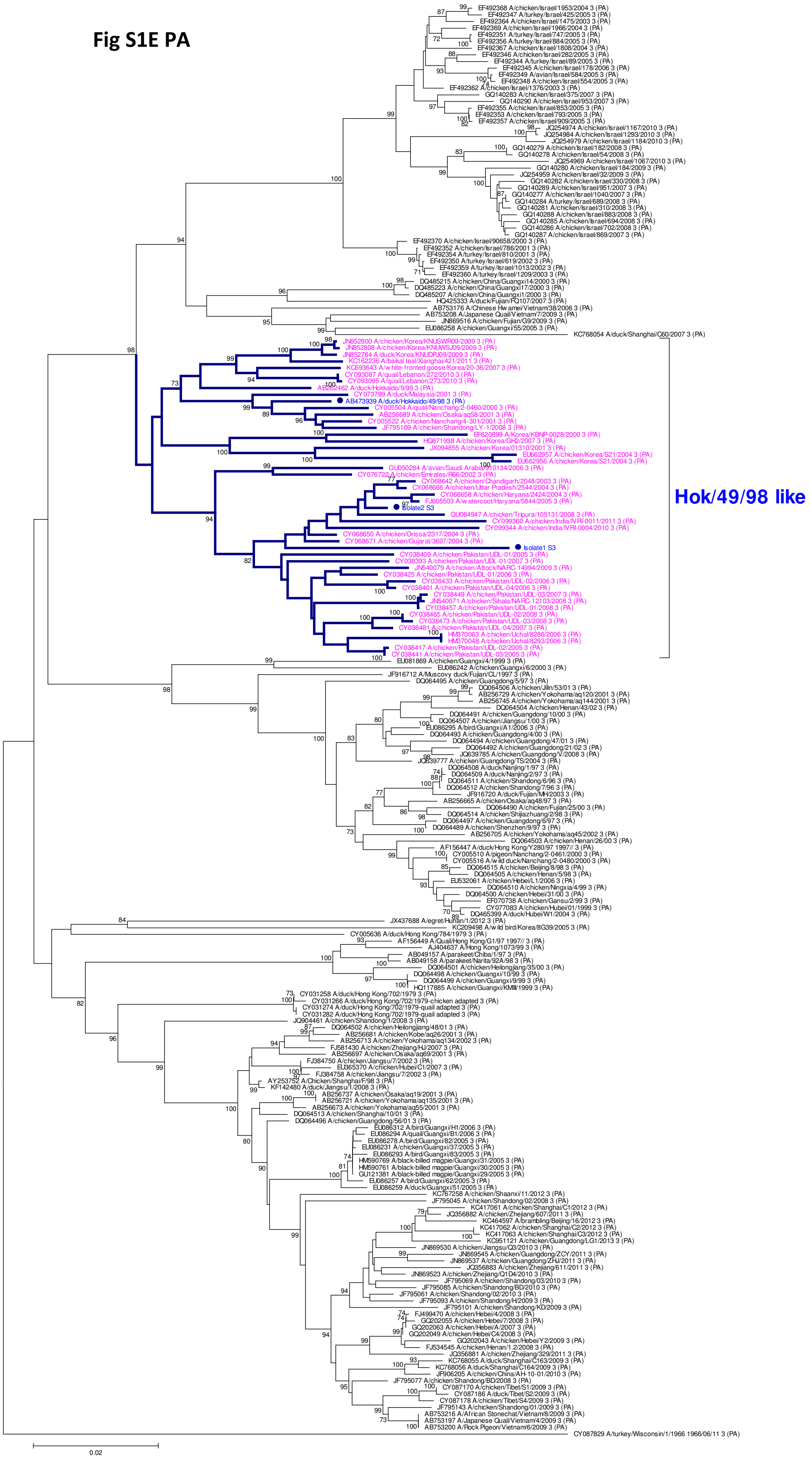
EF492368 A/chicken/Israel/1953/2004 3 (PA)
EF492347 A/turkey/Israel/425/2005 3 (PA)
EF492364 A/chicken/Israel/1475/2003 3 (PA)
EF492369 A/chicken/Israel/1966/2004 3 (PA)
EF492351 A/turkey/Israel/747/2005 3 (PA)
EF492356 A/turkey/Israel/884/2005 3 (PA)
EF492367 A/chicken/Israel/1808/2004 3 (PA)
EF492346 A/chicken/Israel/282/2005 3 (PA)
EF492344 A/turkey/Israel/89/2005 3 (PA)
EF492345 A/chicken/Israel/178/2006 3 (PA)
EF492349 A/avian/Israel/584/2005 3 (PA)
EF492348 A/chicken/Israel/554/2005 3 (PA)
EF492362 A/chicken/Israel/1376/2003 3 (PA)
GQ140283 A/chicken/Israel/375/2007 3 (PA)
GQ140290 A/chicken/Israel/953/2007 3 (PA)
EF492355 A/chicken/Israel/853/2005 3 (PA)
EF492353 A/chicken/Israel/793/2005 3 (PA)
EF492357 A/chicken/Israel/909/2005 3 (PA)
JQ254974 A/chicken/Israel/1167/2010 3 (PA)
JQ254984 A/chicken/Israel/1293/2010 3 (PA)
JQ254979 A/chicken/Israel/1184/2010 3 (PA)
GQ140279 A/chicken/Israel/182/2008 3 (PA)
GQ140278 A/chicken/Israel/54/2008 3 (PA)
JQ254969 A/chicken/Israel/1067/2010 3 (PA)
GQ140280 A/chicken/Israel/184/2009 3 (PA)
JQ254959 A/chicken/Israel/32/2009 3 (PA)
GQ140282 A/chicken/Israel/330/2008 3 (PA)
GQ140289 A/chicken/Israel/951/2007 3 (PA)
GQ140277 A/chicken/Israel/1040/2007 3 (PA)
GQ140284 A/turkey/Israel/689/2008 3 (PA)
GQ140281 A/chicken/Israel/310/2008 3 (PA)
GQ140288 A/chicken/Israel/883/2008 3 (PA)
GQ140285 A/chicken/Israel/694/2008 3 (PA)
GQ140286 A/chicken/Israel/702/2008 3 (PA)
GQ140287 A/chicken/Israel/869/2007 3 (PA)
EF492370 A/chicken/Israel/90658/2000 3 (PA)
EF492352 A/chicken/Israel/786/2001 3 (PA)
EF492354 A/turkey/Israel/810/2001 3 (PA)
EF492350 A/turkey/Israel/619/2002 3 (PA)
EF492359 A/turkey/Israel/1013/2002 3 (PA)
EF492360 A/turkey/Israel/1209/2003 3 (PA)
DQ485215 A/chicken/China/Guangxi14/2000 3 (PA)
DQ485223 A/chicken/China/Guangxi17/2000 3 (PA)
DQ485207 A/chicken/China/Guangxi1/2000 3 (PA)
HQ425333 A/duck/Fujian/FQ107/2007 3 (PA)
AB753176 A/Chinese Hwamei/Vietnam/38/2006 3 (PA)
AB753208 A/Japanese Quail/Vietnam/7/2009 3 (PA)
JN869516 A/chicken/Fujian/G9/2009 3 (PA)
EU086258 A/chicken/Guangxi/55/2005 3 (PA)
KC768054 A/duck/Shanghai/C60/2007 3 (PA)
JN852800 A/chicken/Korea/KNUSWR09/2009 3 (PA)
JN852808 A/chicken/Korea/KNUWSJ09/2009 3 (PA)
JN852784 A/duck/Korea/KNUDPJ09/2009 3 (PA)
KC162236 A/baikal teal/Xianghai/421/2011 3 (PA)
KC693643 A/white-fronted goose/Korea/20-36/2007 3 (PA)
CY093087 A/quail/Lebanon/272/2010 3 (PA)
CY093095 A/quail/Lebanon/273/2010 3 (PA)
AB262462 A/duck/Hokkaido/9/99 3 (PA)
CY073799 A/duck/Malaysia/2001 3 (PA)
AB473939 A/duck/Hokkaido/49/98 3 (PA)
CY005504 A/quail/Nanchang/2-0460/2000 3 (PA)
AB256689 A/chicken/Osaka/aq58/2001 3 (PA)
CY005522 A/chicken/Nanchang/4-301/2001 3 (PA)
JF795109 A/chicken/Shandong/LY-1/2008 3 (PA)
EF620899 A/Korea/KBNP-0028/2000 3 (PA)
HQ871938 A/chicken/Korea/GH2/2007 3 (PA)
JX094855 A/chicken/Korea/01310/2001 3 (PA)
EU662957 A/chicken/Korea/S21/2004 3 (PA)
EU662958 A/chicken/Korea/S21/2004 3 (PA)
GU050284 A/avian/Saudi Arabia/910134/2006 3 (PA)
CY076727 A/chicken/Emirates/R66/2002 3 (PA)
CY068642 A/chicken/Chandigarh/2048/2003 3 (PA)
CY068666 A/chicken/Uttar Pradesh/2544/2004 3 (PA)
CY068658 A/chicken/Haryana/2424/2004 3 (PA)
FJ605503 A/watercoot/Haryana/5844/2005 3 (PA)
Isolate2 S3
GU064947 A/chicken/Tripura/105131/2008 3 (PA)
CY099360 A/chicken/India/IVRI-0011/2011 3 (PA)
CY099344 A/chicken/India/IVRI-0004/2010 3 (PA)
CY068650 A/chicken/Orissa/2317/2004 3 (PA)
CY068671 A/chicken/Gujarat/3697/2004 3 (PA)
Isolate1 S3
CY038409 A/chicken/Pakistan/UDL-01/2005 3 (PA)
CY038393 A/chicken/Pakistan/UDL-01/2007 3 (PA)
JN540079 A/chicken/Attock/NARC-14994/2009 3 (PA)
CY038425 A/chicken/Pakistan/UDL-01/2006 3 (PA)
CY038433 A/chicken/Pakistan/UDL-02/2006 3 (PA)
CY038401 A/chicken/Pakistan/UDL-04/2006 3 (PA)
CY038449 A/chicken/Pakistan/UDL-03/2007 3 (PA)
JN540071 A/chicken/Sihala/NARC-12103/2008 3 (PA)
CY038457 A/chicken/Pakistan/UDL-01/2008 3 (PA)
CY038465 A/chicken/Pakistan/UDL-02/2008 3 (PA)
CY038473 A/chicken/Pakistan/UDL-03/2008 3 (PA)
CY038481 A/chicken/Pakistan/UDL-04/2007 3 (PA)
HM370063 A/chicken/Uchal/8286/2006 3 (PA)
HM370048 A/chicken/Uchal/8293/2006 3 (PA)
CY038417 A/chicken/Pakistan/UDL-02/2005 3 (PA)
CY038441 A/chicken/Pakistan/UDL-03/2005 3 (PA)
Hok/49/98 like
EU081869 A/chicken/Guangxi/4/1999 3 (PA)
EU086242 A/chicken/Guangxi/6/2000 3 (PA)
JF916712 A/Muscovy duck/Fujian/CL/1997 3 (PA)
DQ064495 A/chicken/Guangdong/5/97 3 (PA)
DQ064506 A/chicken/Jilin/53/01 3 (PA)
AB256729 A/chicken/Yokohama/aq120/2001 3 (PA)
AB256745 A/chicken/Yokohama/aq144/2001 3 (PA)
DQ064504 A/chicken/Henan/43/02 3 (PA)
DQ064491 A/chicken/Guangdong/10/00 3 (PA)
DQ064507 A/chicken/Jiangsu/1/00 3 (PA)
EU086295 A/bird/Guangxi/A1/2006 3 (PA)
DQ064493 A/chicken/Guangdong/4/00 3 (PA)
DQ064494 A/chicken/Guangdong/47/01 3 (PA)
DQ064492 A/chicken/Guangdong/21/02 3 (PA)
JQ639785 A/chicken/Guangdong/V/2008 3 (PA)
JQ639777 A/chicken/Guangdong/TS/2004 3 (PA)
DQ064508 A/duck/Nanjing/1/97 3 (PA)
DQ064509 A/duck/Nanjing/2/97 3 (PA)
DQ064511 A/chicken/Shandong/6/96 3 (PA)
DQ064512 A/chicken/Shandong/7/96 3 (PA)
JF916720 A/duck/Fujian/MH/2003 3 (PA)
AB256665 A/chicken/Osaka/aq48/97 3 (PA)
DQ064490 A/chicken/Fujian/25/00 3 (PA)
DQ064514 A/chicken/Shijiazhuang/2/98 3 (PA)
DQ064497 A/chicken/Guangdong/6/97 3 (PA)
DQ064489 A/chicken/Shenzhen/9/97 3 (PA)
AB256705 A/chicken/Yokohama/aq45/2002 3 (PA)
DQ064503 A/chicken/Henan/26/00 3 (PA)
AF156447 A/duck/Hong Kong/Y280/97 1997// 3 (PA)
CY005510 A/pigeon/Nanchang/2-0461/2000 3 (PA)
CY005516 A/wild duck/Nanchang/2-0480/2000 3 (PA)
DQ064515 A/chicken/Beijing/8/98 3 (PA)
DQ064505 A/chicken/Henan/5/98 3 (PA)
EU532061 A/chicken/Hebei/L1/2006 3 (PA)
DQ064510 A/chicken/Ningxia/4/99 3 (PA)
DQ064500 A/chicken/Hebei/31/00 3 (PA)
EF070738 A/chicken/Gansu/2/99 3 (PA)
CY077083 A/chicken/Hubei/01/1999 3 (PA)
DQ465399 A/duck/Hubei/W1/2004 3 (PA)
JX437688 A/egret/Hunan/1/2012 3 (PA)
KC209498 A/wild bird/Korea/8G39/2005 3 (PA)
CY005636 A/duck/Hong Kong/784/1979 3 (PA)
AF156449 A/Quail/Hong Kong/G1/97 1997// 3 (PA)
AJ404637 A/Hong Kong/1073/99 3 (PA)
AB049157 A/parakeet/Chiba/1/97 3 (PA)
AB049158 A/parakeet/Narita/92A/98 3 (PA)
DQ064501 A/chicken/Heilongjiang/35/00 3 (PA)
DQ064498 A/chicken/Guangxi/10/99 3 (PA)
DQ064499 A/chicken/Guangxi/9/99 3 (PA)
HQ117885 A/chicken/Guangxi/KMII/1999 3 (PA)
CY031258 A/duck/Hong Kong/702/1979 3 (PA)
CY031266 A/duck/Hong Kong/702/1979-chicken adapted 3 (PA)
CY031274 A/duck/Hong Kong/702/1979-quail adapted 3 (PA)
CY031282 A/duck/Hong Kong/702/1979-quail adapted 3 (PA)
JQ904461 A/chicken/Shandong/1/2008 3 (PA)
DQ064502 A/chicken/Heilongjiang/48/01 3 (PA)
AB256681 A/chicken/Kobe/aq26/2001 3 (PA)
AB256713 A/chicken/Yokohama/aq134/2002 3 (PA)
FJ581430 A/chicken/Zhejiang/HJ/2007 3 (PA)
AB256697 A/chicken/Osaka/aq69/2001 3 (PA)
FJ384750 A/chicken/Jiangsu/7/2002 3 (PA)
EU365370 A/chicken/Hubei/C1/2007 3 (PA)
FJ384758 A/chicken/Jiangsu/7/2002 3 (PA)
AY253752 A/Chicken/Shanghai/F/98 3 (PA)
KF142480 A/duck/Jiangsu/1/2008 3 (PA)
AB256737 A/chicken/Osaka/aq19/2001 3 (PA)
AB256721 A/chicken/Yokohama/aq135/2001 3 (PA)
AB256673 A/chicken/Yokohama/aq55/2001 3 (PA)
DQ064513 A/chicken/Shanghai/10/01 3 (PA)
DQ064496 A/chicken/Guangdong/56/01 3 (PA)
EU086312 A/bird/Guangxi/H1/2006 3 (PA)
EU086294 A/quail/Guangxi/B1/2006 3 (PA)
EU086278 A/bird/Guangxi/82/2005 3 (PA)
EU086231 A/chicken/Guangxi/37/2005 3 (PA)
EU086293 A/bird/Guangxi/83/2005 3 (PA)
HM590769 A/black-billed magpie/Guangxi/31/2005 3 (PA)
HM590761 A/black-billed magpie/Guangxi/30/2005 3 (PA)
GU121381 A/black-billed magpie/Guangxi/29/2005 3 (PA)
EU086257 A/bird/Guangxi/62/2005 3 (PA)
EU086259 A/duck/Guangxi/51/2005 3 (PA)
KC767258 A/chicken/Shaanxi/11/2012 3 (PA)
JF795045 A/chicken/Shandong/02/2008 3 (PA)
KC417061 A/chicken/Shanghai/C1/2012 3 (PA)
JQ356882 A/chicken/Zhejiang/607/2011 3 (PA)
KC464597 A/brambling/Beijing/16/2012 3 (PA)
KC417062 A/chicken/Shanghai/C2/2012 3 (PA)
KC417063 A/chicken/Shanghai/C3/2012 3 (PA)
KC951121 A/chicken/Guangdong/LG1/2013 3 (PA)
JN869530 A/chicken/Jiangsu/Q3/2010 3 (PA)
JN869545 A/chicken/Guangdong/ZCY/2011 3 (PA)
JN869537 A/chicken/Guangdong/ZHJ/2011 3 (PA)
JQ356883 A/chicken/Zhejiang/611/2011 3 (PA)
JN869523 A/chicken/Zhejiang/Q1D4/2010 3 (PA)
JF795069 A/chicken/Shandong/03/2010 3 (PA)
JF795085 A/chicken/Shandong/BD/2010 3 (PA)
JF795061 A/chicken/Shandong/02/2010 3 (PA)
JF795093 A/chicken/Shandong/H/2009 3 (PA)
JF795101 A/chicken/Shandong/KD/2009 3 (PA)
FJ499470 A/chicken/Hebei/4/2008 3 (PA)
GQ202055 A/chicken/Hebei/7/2008 3 (PA)
GQ202063 A/chicken/Hebei/A/2007 3 (PA)
GQ202049 A/chicken/Hebei/C4/2008 3 (PA)
GQ202043 A/chicken/Hebei/Y2/2009 3 (PA)
FJ534545 A/chicken/Henan/1.2/2008 3 (PA)
JQ356881 A/chicken/Zhejiang/329/2011 3 (PA)
KC768055 A/duck/Shanghai/C163/2009 3 (PA)
KC768056 A/duck/Shanghai/C164/2009 3 (PA)
JF906205 A/chicken/China/AH-10-01/2010 3 (PA)
JF795077 A/chicken/Shandong/BD/2008 3 (PA)
CY087170 A/chicken/Tibet/S1/2009 3 (PA)
CY087186 A/duck/Tibet/S2/2009 3 (PA)
CY087178 A/chicken/Tibet/S4/2009 3 (PA)
JF795143 A/chicken/Shandong/01/2009 3 (PA)
AB753216 A/African Stonechat/Vietnam/8/2009 3 (PA)
AB753197 A/Japanese Quail/Vietnam/4/2009 3 (PA)
AB753200 A/Rock Pigeon/Vietnam/6/2009 3 (PA)
CY087829 A/turkey/Wisconsin/1/1966 1966/06/11 3 (PA)
0.02

Fig S1F NP

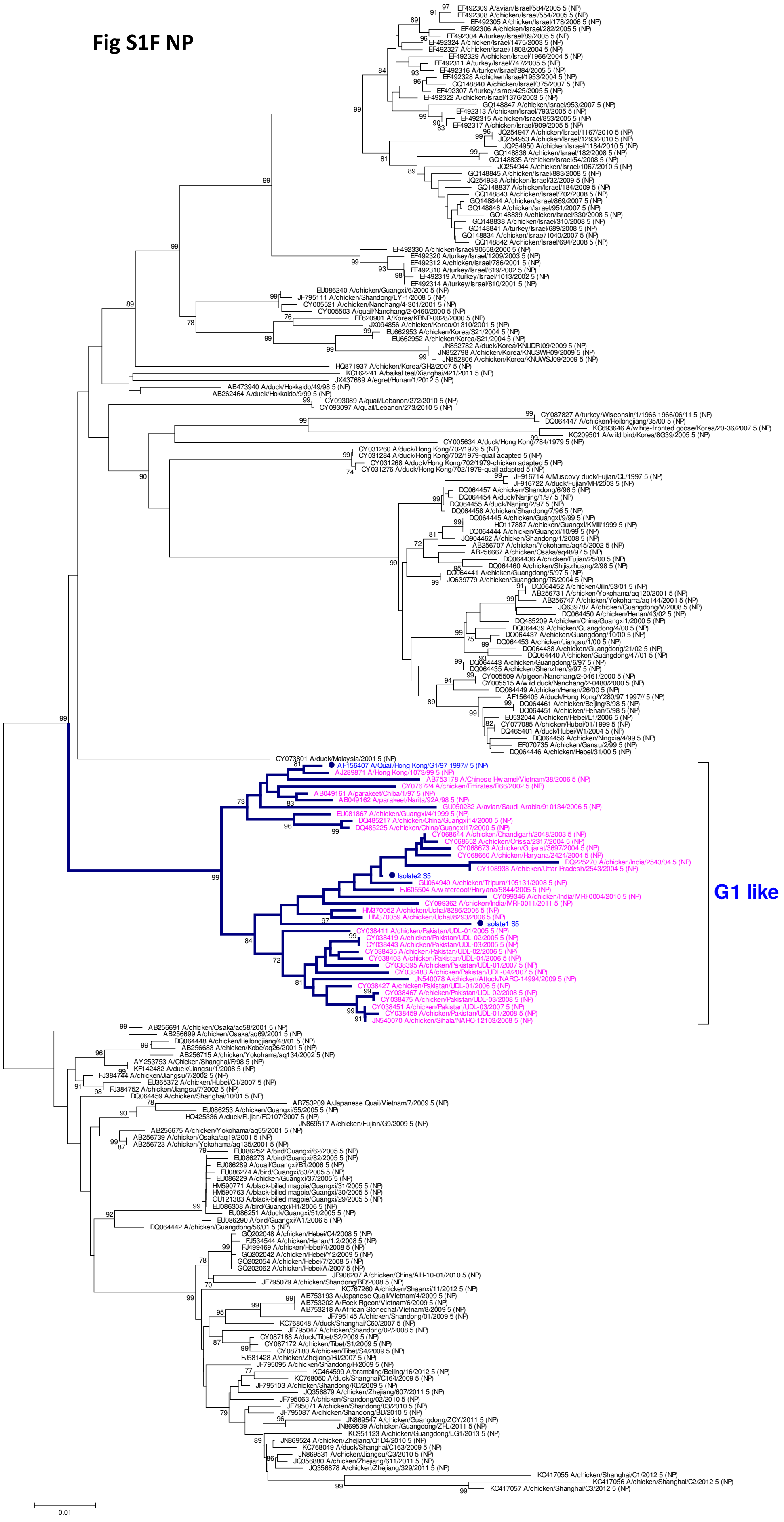
EF492309 A/avian/Israel/584/2005 5 (NP)
EF492308 A/chicken/Israel/554/2005 5 (NP)
EF492305 A/chicken/Israel/178/2006 5 (NP)
EF492306 A/chicken/Israel/282/2005 5 (NP)
EF492304 A/turkey/Israel/89/2005 5 (NP)
EF492324 A/chicken/Israel/1475/2003 5 (NP)
EF492327 A/chicken/Israel/1808/2004 5 (NP)
EF492329 A/chicken/Israel/1966/2004 5 (NP)
EF492311 A/turkey/Israel/747/2005 5 (NP)
EF492316 A/turkey/Israel/884/2005 5 (NP)
EF492328 A/chicken/Israel/1953/2004 5 (NP)
GQ148840 A/chicken/Israel/375/2007 5 (NP)
EF492307 A/turkey/Israel/425/2005 5 (NP)
EF492322 A/chicken/Israel/1376/2003 5 (NP)
GQ148847 A/chicken/Israel/953/2007 5 (NP)
EF492313 A/chicken/Israel/793/2005 5 (NP)
EF492315 A/chicken/Israel/853/2005 5 (NP)
EF492317 A/chicken/Israel/909/2005 5 (NP)
JQ254947 A/chicken/Israel/1167/2010 5 (NP)
JQ254953 A/chicken/Israel/1293/2010 5 (NP)
JQ254950 A/chicken/Israel/1184/2010 5 (NP)
GQ148836 A/chicken/Israel/182/2008 5 (NP)
GQ148835 A/chicken/Israel/54/2008 5 (NP)
JQ254944 A/chicken/Israel/1067/2010 5 (NP)
GQ148845 A/chicken/Israel/883/2008 5 (NP)
JQ254938 A/chicken/Israel/32/2009 5 (NP)
GQ148837 A/chicken/Israel/184/2009 5 (NP)
GQ148843 A/chicken/Israel/702/2008 5 (NP)
GQ148844 A/chicken/Israel/869/2007 5 (NP)
GQ148846 A/chicken/Israel/951/2007 5 (NP)
GQ148839 A/chicken/Israel/330/2008 5 (NP)
GQ148838 A/chicken/Israel/310/2008 5 (NP)
GQ148841 A/turkey/Israel/689/2008 5 (NP)
GQ148834 A/chicken/Israel/1040/2007 5 (NP)
GQ148842 A/chicken/Israel/694/2008 5 (NP)
EF492330 A/chicken/Israel/90658/2000 5 (NP)
EF492320 A/turkey/Israel/1209/2003 5 (NP)
EF492312 A/chicken/Israel/786/2001 5 (NP)
EF492310 A/turkey/Israel/619/2002 5 (NP)
EF492319 A/turkey/Israel/1013/2002 5 (NP)
EF492314 A/turkey/Israel/810/2001 5 (NP)
EU086240 A/chicken/Guangxi/6/2000 5 (NP)
JF795111 A/chicken/Shandong/LY-1/2008 5 (NP)
CY005521 A/chicken/Nanchang/4-301/2001 5 (NP)
CY005503 A/quail/Nanchang/2-0460/2000 5 (NP)
EF620901 A/Korea/KBNP-0028/2000 5 (NP)
JX094856 A/chicken/Korea/01310/2001 5 (NP)
EU662953 A/chicken/Korea/S21/2004 5 (NP)
EU662952 A/chicken/Korea/S21/2004 5 (NP)
JN852782 A/duck/Korea/KNUDPJ09/2009 5 (NP)
JN852798 A/chicken/Korea/KNUSWR09/2009 5 (NP)
JN852806 A/chicken/Korea/KNUWSJ09/2009 5 (NP)
HQ871937 A/chicken/Korea/GH2/2007 5 (NP)
KC162241 A/baikal teal/Xianghai/421/2011 5 (NP)
JX437689 A/egret/Hunan/1/2012 5 (NP)
AB473940 A/duck/Hokkaido/49/98 5 (NP)
AB262464 A/duck/Hokkaido/9/99 5 (NP)
CY093089 A/quail/Lebanon/272/2010 5 (NP)
CY093097 A/quail/Lebanon/273/2010 5 (NP)
CY087827 A/turkey/Wisconsin/1/1966 1966/06/11 5 (NP)
DQ064447 A/chicken/Heilongjiang/35/00 5 (NP)
KC693646 A/white-fronted goose/Korea/20-36/2007 5 (NP)
KC209501 A/wild bird/Korea/8G39/2005 5 (NP)
CY005634 A/duck/Hong Kong/784/1979 5 (NP)
CY031260 A/duck/Hong Kong/702/1979 5 (NP)
CY031284 A/duck/Hong Kong/702/1979-quail adapted 5 (NP)
CY031268 A/duck/Hong Kong/702/1979-chicken adapted 5 (NP)
CY031276 A/duck/Hong Kong/702/1979-quail adapted 5 (NP)
JF916714 A/Muscovy duck/Fujian/CL/1997 5 (NP)
JF916722 A/duck/Fujian/MH/2003 5 (NP)
DQ064457 A/chicken/Shandong/6/96 5 (NP)
DQ064454 A/duck/Nanjing/1/97 5 (NP)
DQ064455 A/duck/Nanjing/2/97 5 (NP)
DQ064458 A/chicken/Shandong/7/96 5 (NP)
DQ064445 A/chicken/Guangxi/9/99 5 (NP)
HQ117887 A/chicken/Guangxi/KMIII/1999 5 (NP)
DQ064444 A/chicken/Guangxi/10/99 5 (NP)
JQ904462 A/chicken/Shandong/1/2008 5 (NP)
AB256707 A/chicken/Yokohama/aq45/2002 5 (NP)
AB256667 A/chicken/Osaka/aq48/97 5 (NP)
DQ064436 A/chicken/Fujian/25/00 5 (NP)
DQ064460 A/chicken/Shijiazhuang/2/98 5 (NP)
DQ064441 A/chicken/Guangdong/5/97 5 (NP)
JQ639779 A/chicken/Guangdong/TS/2004 5 (NP)
DQ064452 A/chicken/Jilin/53/01 5 (NP)
AB256731 A/chicken/Yokohama/aq120/2001 5 (NP)
AB256747 A/chicken/Yokohama/aq144/2001 5 (NP)
JQ639787 A/chicken/Guangdong/V/2008 5 (NP)
DQ064450 A/chicken/Henan/43/02 5 (NP)
DQ485209 A/chicken/China/Guangxi1/2000 5 (NP)
DQ064439 A/chicken/Guangdong/4/00 5 (NP)
DQ064437 A/chicken/Guangdong/10/00 5 (NP)
DQ064453 A/chicken/Jiangsu/1/00 5 (NP)
DQ064438 A/chicken/Guangdong/21/02 5 (NP)
DQ064440 A/chicken/Guangdong/47/01 5 (NP)
DQ064443 A/chicken/Guangdong/6/97 5 (NP)
DQ064435 A/chicken/Shenzhen/9/97 5 (NP)
CY005509 A/pigeon/Nanchang/2-0461/2000 5 (NP)
CY005515 A/wild duck/Nanchang/2-0480/2000 5 (NP)
DQ064449 A/chicken/Henan/26/00 5 (NP)
AF156405 A/duck/Hong Kong/Y280/97 1997// 5 (NP)
DQ064461 A/chicken/Beijing/8/98 5 (NP)
DQ064451 A/chicken/Henan/5/98 5 (NP)
EU532044 A/chicken/Hebei/L1/2006 5 (NP)
CY077085 A/chicken/Hubei/01/1999 5 (NP)
DQ465401 A/duck/Hubei/W1/2004 5 (NP)
DQ064456 A/chicken/Ningxia/4/99 5 (NP)
EF070735 A/chicken/Gansu/2/99 5 (NP)
DQ064446 A/chicken/Hebei/31/00 5 (NP)
CY073801 A/duck/Malaysia/2001 5 (NP)
AF156407 A/Quail/Hong Kong/G1/97 1997// 5 (NP)
AJ289871 A/Hong Kong/1073/99 5 (NP)
AB753178 A/Chinese Hwamei/Vietnam/38/2006 5 (NP)
CY076724 A/chicken/Emirates/R66/2002 5 (NP)
AB049161 A/parakeet/Chiba/1/97 5 (NP)
AB049162 A/parakeet/Narita/92A/98 5 (NP)
GU050282 A/avian/Saudi Arabia/910134/2006 5 (NP)
EU081867 A/chicken/Guangxi/4/1999 5 (NP)
DQ485217 A/chicken/China/Guangxi14/2000 5 (NP)
DQ485225 A/chicken/China/Guangxi17/2000 5 (NP)
CY068644 A/chicken/Chandigarh/2048/2003 5 (NP)
CY068652 A/chicken/Orissa/2317/2004 5 (NP)
CY068673 A/chicken/Gujarat/3697/2004 5 (NP)
CY068660 A/chicken/Haryana/2424/2004 5 (NP)
DQ225270 A/chicken/India/2543/04 5 (NP)
CY108938 A/chicken/Uttar Pradesh/2543/2004 5 (NP)
Isolate2 S5
GU064949 A/chicken/Tripura/105131/2008 5 (NP)
FJ605504 A/watercoot/Haryana/5844/2005 5 (NP)
CY099346 A/chicken/India/IVRI-0004/2010 5 (NP)
CY099362 A/chicken/India/IVRI-0011/2011 5 (NP)
HM370052 A/chicken/Uchal/8286/2006 5 (NP)
HM370059 A/chicken/Uchal/8293/2006 5 (NP)
Isolate1 S5
CY038411 A/chicken/Pakistan/UDL-01/2005 5 (NP)
CY038419 A/chicken/Pakistan/UDL-02/2005 5 (NP)
CY038443 A/chicken/Pakistan/UDL-03/2005 5 (NP)
CY038435 A/chicken/Pakistan/UDL-02/2006 5 (NP)
CY038403 A/chicken/Pakistan/UDL-04/2006 5 (NP)
CY038395 A/chicken/Pakistan/UDL-01/2007 5 (NP)
CY038483 A/chicken/Pakistan/UDL-04/2007 5 (NP)
JN540078 A/chicken/Attock/NARC-14994/2009 5 (NP)
CY038427 A/chicken/Pakistan/UDL-01/2006 5 (NP)
CY038467 A/chicken/Pakistan/UDL-02/2008 5 (NP)
CY038475 A/chicken/Pakistan/UDL-03/2008 5 (NP)
CY038451 A/chicken/Pakistan/UDL-03/2007 5 (NP)
CY038459 A/chicken/Pakistan/UDL-01/2008 5 (NP)
JN540070 A/chicken/Sihala/NARC-12103/2008 5 (NP)
G1 like
AB256691 A/chicken/Osaka/aq58/2001 5 (NP)
AB256699 A/chicken/Osaka/aq69/2001 5 (NP)
DQ064448 A/chicken/Heilongjiang/48/01 5 (NP)
AB256683 A/chicken/Kobe/aq26/2001 5 (NP)
AB256715 A/chicken/Yokohama/aq134/2002 5 (NP)
AY253753 A/Chicken/Shanghai/F/98 5 (NP)
KF142482 A/duck/Jiangsu/1/2008 5 (NP)
FJ384744 A/chicken/Jiangsu/7/2002 5 (NP)
EU365372 A/chicken/Hubei/C1/2007 5 (NP)
FJ384752 A/chicken/Jiangsu/7/2002 5 (NP)
DQ064459 A/chicken/Shanghai/10/01 5 (NP)
AB753209 A/Japanese Quail/Vietnam/7/2009 5 (NP)
EU086253 A/chicken/Guangxi/55/2005 5 (NP)
HQ425336 A/duck/Fujian/FQ107/2007 5 (NP)
JN869517 A/chicken/Fujian/G9/2009 5 (NP)
AB256675 A/chicken/Yokohama/aq55/2001 5 (NP)
AB256739 A/chicken/Osaka/aq19/2001 5 (NP)
AB256723 A/chicken/Yokohama/aq135/2001 5 (NP)
EU086252 A/bird/Guangxi/62/2005 5 (NP)
EU086273 A/bird/Guangxi/82/2005 5 (NP)
EU086289 A/quail/Guangxi/B1/2006 5 (NP)
EU086274 A/bird/Guangxi/83/2005 5 (NP)
EU086229 A/chicken/Guangxi/37/2005 5 (NP)
HM590771 A/black-billed magpie/Guangxi/31/2005 5 (NP)
HM590763 A/black-billed magpie/Guangxi/30/2005 5 (NP)
GU121383 A/black-billed magpie/Guangxi/29/2005 5 (NP)
EU086308 A/bird/Guangxi/H1/2006 5 (NP)
EU086251 A/duck/Guangxi/51/2005 5 (NP)
EU086290 A/bird/Guangxi/A1/2006 5 (NP)
DQ064442 A/chicken/Guangdong/56/01 5 (NP)
GQ202048 A/chicken/Hebei/C4/2008 5 (NP)
FJ534544 A/chicken/Henan/1.2/2008 5 (NP)
FJ499469 A/chicken/Hebei/4/2008 5 (NP)
GQ202042 A/chicken/Hebei/Y2/2009 5 (NP)
GQ202054 A/chicken/Hebei/7/2008 5 (NP)
GQ202062 A/chicken/Hebei/A/2007 5 (NP)
JF906207 A/chicken/China/AH-10-01/2010 5 (NP)
JF795079 A/chicken/Shandong/BD/2008 5 (NP)
KC767260 A/chicken/Shaanxi/11/2012 5 (NP)
AB753193 A/Japanese Quail/Vietnam/4/2009 5 (NP)
AB753202 A/Rock Pigeon/Vietnam/6/2009 5 (NP)
AB753218 A/African Stonechat/Vietnam/8/2009 5 (NP)
JF795145 A/chicken/Shandong/01/2009 5 (NP)
KC768048 A/duck/Shanghai/C60/2007 5 (NP)
JF795047 A/chicken/Shandong/02/2008 5 (NP)
CY087188 A/duck/Tibet/S2/2009 5 (NP)
CY087172 A/chicken/Tibet/S1/2009 5 (NP)
CY087180 A/chicken/Tibet/S4/2009 5 (NP)
FJ581428 A/chicken/Zhejiang/HJ/2007 5 (NP)
JF795095 A/chicken/Shandong/H/2009 5 (NP)
KC464599 A/brambling/Beijing/16/2012 5 (NP)
KC768050 A/duck/Shanghai/C164/2009 5 (NP)
JF795103 A/chicken/Shandong/KD/2009 5 (NP)
JQ356879 A/chicken/Zhejiang/607/2011 5 (NP)
JF795063 A/chicken/Shandong/02/2010 5 (NP)
JF795071 A/chicken/Shandong/03/2010 5 (NP)
JF795087 A/chicken/Shandong/BD/2010 5 (NP)
JN869547 A/chicken/Guangdong/ZCY/2011 5 (NP)
JN869539 A/chicken/Guangdong/ZHJ/2011 5 (NP)
KC951123 A/chicken/Guangdong/LG1/2013 5 (NP)
JN869524 A/chicken/Zhejiang/Q1D4/2010 5 (NP)
KC768049 A/duck/Shanghai/C163/2009 5 (NP)
JN869531 A/chicken/Jiangsu/Q3/2010 5 (NP)
JQ356880 A/chicken/Zhejiang/611/2011 5 (NP)
JQ356878 A/chicken/Zhejiang/329/2011 5 (NP)
KC417055 A/chicken/Shanghai/C1/2012 5 (NP)
KC417056 A/chicken/Shanghai/C2/2012 5 (NP)
KC417057 A/chicken/Shanghai/C3/2012 5 (NP)
0.01

Fig S1G MP

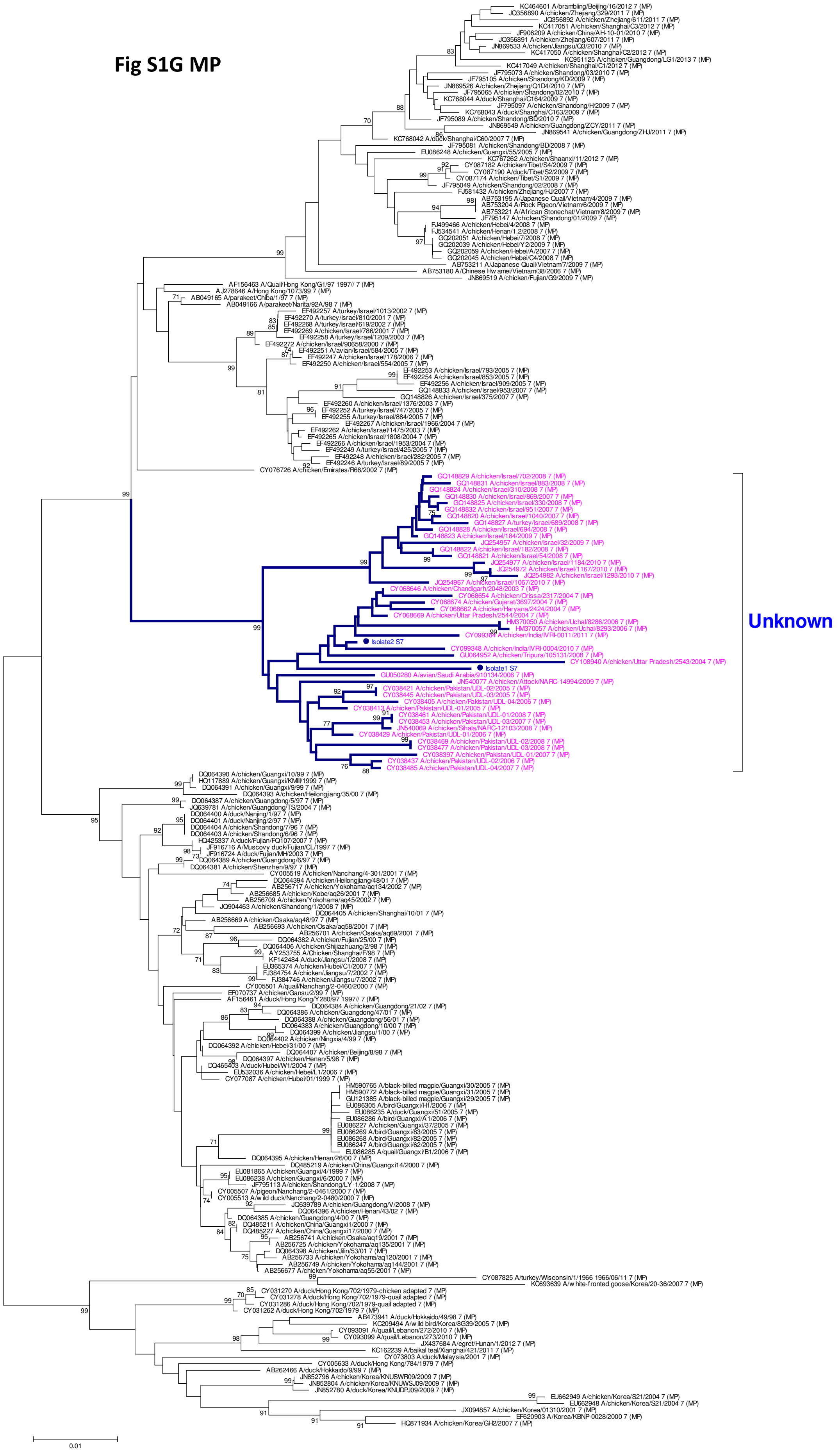

Fig S1H NS

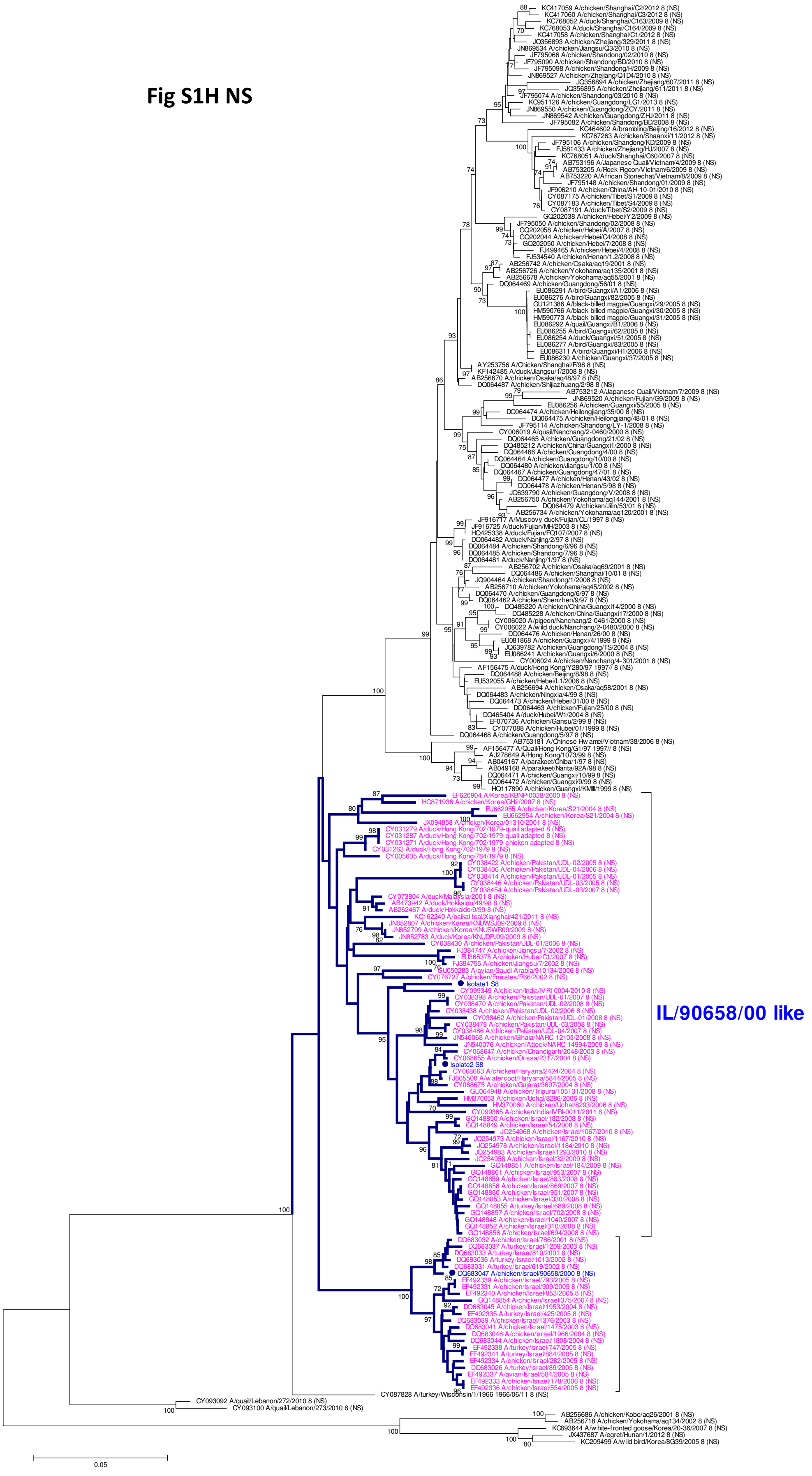
KC417059 A/chicken/Shanghai/C2/2012 8 (NS)
KC417060 A/chicken/Shanghai/C3/2012 8 (NS)
KC768052 A/duck/Shanghai/C163/2009 8 (NS)
KC768053 A/duck/Shanghai/C164/2009 8 (NS)
KC417058 A/chicken/Shanghai/C1/2012 8 (NS)
JQ356893 A/chicken/Zhejiang/329/2011 8 (NS)
JN869534 A/chicken/Jiangsu/Q3/2010 8 (NS)
JF795066 A/chicken/Shandong/02/2010 8 (NS)
JF795090 A/chicken/Shandong/BD/2010 8 (NS)
JF795098 A/chicken/Shandong/H/2009 8 (NS)
JN869527 A/chicken/Zhejiang/Q1D4/2010 8 (NS)
JQ356894 A/chicken/Zhejiang/607/2011 8 (NS)
JQ356895 A/chicken/Zhejiang/611/2011 8 (NS)
JF795074 A/chicken/Shandong/03/2010 8 (NS)
KC951126 A/chicken/Guangdong/LG1/2013 8 (NS)
JN869550 A/chicken/Guangdong/ZCY/2011 8 (NS)
JN869542 A/chicken/Guangdong/ZHJ/2011 8 (NS)
JF795082 A/chicken/Shandong/BD/2008 8 (NS)
KC464602 A/brambling/Beijing/16/2012 8 (NS)
KC767263 A/chicken/Shaanxi/11/2012 8 (NS)
JF795106 A/chicken/Shandong/KD/2009 8 (NS)
FJ581433 A/chicken/Zhejiang/HJ/2007 8 (NS)
KC768051 A/duck/Shanghai/C60/2007 8 (NS)
AB753196 A/Japanese Quail/Vietnam/4/2009 8 (NS)
AB753205 A/Rock Pigeon/Vietnam/6/2009 8 (NS)
AB753220 A/African Stonechat/Vietnam/8/2009 8 (NS)
JF795148 A/chicken/Shandong/01/2009 8 (NS)
JF906210 A/chicken/China/AH-10-01/2010 8 (NS)
CY087175 A/chicken/Tibet/S1/2009 8 (NS)
CY087183 A/chicken/Tibet/S4/2009 8 (NS)
CY087191 A/duck/Tibet/S2/2009 8 (NS)
GQ202038 A/chicken/Hebei/Y2/2009 8 (NS)
JF795050 A/chicken/Shandong/02/2008 8 (NS)
GQ202058 A/chicken/Hebei/A/2007 8 (NS)
GQ202044 A/chicken/Hebei/C4/2008 8 (NS)
GQ202050 A/chicken/Hebei/7/2008 8 (NS)
FJ499465 A/chicken/Hebei/4/2008 8 (NS)
FJ534540 A/chicken/Henan/1.2/2008 8 (NS)
AB256742 A/chicken/Osaka/aq19/2001 8 (NS)
AB256726 A/chicken/Yokohama/aq135/2001 8 (NS)
AB256678 A/chicken/Yokohama/aq55/2001 8 (NS)
DQ064469 A/chicken/Guangdong/56/01 8 (NS)
EU086291 A/bird/Guangxi/A1/2006 8 (NS)
EU086276 A/bird/Guangxi/82/2005 8 (NS)
GU121386 A/black-billed magpie/Guangxi/29/2005 8 (NS)
HM590766 A/black-billed magpie/Guangxi/30/2005 8 (NS)
HM590773 A/black-billed magpie/Guangxi/31/2005 8 (NS)
EU086292 A/quail/Guangxi/B1/2006 8 (NS)
EU086255 A/bird/Guangxi/62/2005 8 (NS)
EU086254 A/duck/Guangxi/51/2005 8 (NS)
EU086277 A/bird/Guangxi/83/2005 8 (NS)
EU086311 A/bird/Guangxi/H1/2006 8 (NS)
EU086230 A/chicken/Guangxi/37/2005 8 (NS)
AY253756 A/Chicken/Shanghai/F/98 8 (NS)
KF142485 A/duck/Jiangsu/1/2008 8 (NS)
AB256670 A/chicken/Osaka/aq48/97 8 (NS)
DQ064487 A/chicken/Shijiazhuang/2/98 8 (NS)
AB753212 A/Japanese Quail/Vietnam/7/2009 8 (NS)
JN869520 A/chicken/Fujian/G9/2009 8 (NS)
EU086256 A/chicken/Guangxi/55/2005 8 (NS)
DQ064474 A/chicken/Heilongjiang/35/00 8 (NS)
DQ064475 A/chicken/Heilongjiang/48/01 8 (NS)
JF795114 A/chicken/Shandong/LY-1/2008 8 (NS)
CY006019 A/quail/Nanchang/2-0460/2000 8 (NS)
DQ064465 A/chicken/Guangdong/21/02 8 (NS)
DQ485212 A/chicken/China/Guangxi1/2000 8 (NS)
DQ064466 A/chicken/Guangdong/4/00 8 (NS)
DQ064464 A/chicken/Guangdong/10/00 8 (NS)
DQ064480 A/chicken/Jiangsu/1/00 8 (NS)
DQ064467 A/chicken/Guangdong/47/01 8 (NS)
DQ064477 A/chicken/Henan/43/02 8 (NS)
DQ064478 A/chicken/Henan/5/98 8 (NS)
JQ639790 A/chicken/Guangdong/V/2008 8 (NS)
AB256750 A/chicken/Yokohama/aq144/2001 8 (NS)
DQ064479 A/chicken/Jilin/53/01 8 (NS)
AB256734 A/chicken/Yokohama/aq120/2001 8 (NS)
JF916717 A/Muscovy duck/Fujian/CL/1997 8 (NS)
JF916725 A/duck/Fujian/MH/2003 8 (NS)
HQ425338 A/duck/Fujian/FQ107/2007 8 (NS)
DQ064482 A/duck/Nanjing/2/97 8 (NS)
DQ064484 A/chicken/Shandong/6/96 8 (NS)
DQ064485 A/chicken/Shandong/7/96 8 (NS)
DQ064481 A/duck/Nanjing/1/97 8 (NS)
AB256702 A/chicken/Osaka/aq69/2001 8 (NS)
DQ064486 A/chicken/Shanghai/10/01 8 (NS)
JQ904464 A/chicken/Shandong/1/2008 8 (NS)
AB256710 A/chicken/Yokohama/aq45/2002 8 (NS)
DQ064470 A/chicken/Guangdong/6/97 8 (NS)
DQ064462 A/chicken/Shenzhen/9/97 8 (NS)
DQ485220 A/chicken/China/Guangxi14/2000 8 (NS)
DQ485228 A/chicken/China/Guangxi17/2000 8 (NS)
CY006020 A/pigeon/Nanchang/2-0461/2000 8 (NS)
CY006022 A/wild duck/Nanchang/2-0480/2000 8 (NS)
DQ064476 A/chicken/Henan/26/00 8 (NS)
EU081868 A/chicken/Guangxi/4/1999 8 (NS)
JQ639782 A/chicken/Guangdong/TS/2004 8 (NS)
EU086241 A/chicken/Guangxi/6/2000 8 (NS)
CY006024 A/chicken/Nanchang/4-301/2001 8 (NS)
AF156475 A/duck/Hong Kong/Y280/97 1997// 8 (NS)
DQ064488 A/chicken/Beijing/8/98 8 (NS)
EU532055 A/chicken/Hebei/L1/2006 8 (NS)
AB256694 A/chicken/Osaka/aq58/2001 8 (NS)
DQ064483 A/chicken/Ningxia/4/99 8 (NS)
DQ064473 A/chicken/Hebei/31/00 8 (NS)
DQ064463 A/chicken/Fujian/25/00 8 (NS)
DQ465404 A/duck/Hubei/W1/2004 8 (NS)
EF070736 A/chicken/Gansu/2/99 8 (NS)
CY077088 A/chicken/Hubei/01/1999 8 (NS)
DQ064468 A/chicken/Guangdong/5/97 8 (NS)
AB753181 A/Chinese Hwamei/Vietnam/38/2006 8 (NS)
AF156477 A/Quail/Hong Kong/G1/97 1997// 8 (NS)
AJ278649 A/Hong Kong/1073/99 8 (NS)
AB049167 A/parakeet/Chiba/1/97 8 (NS)
AB049168 A/parakeet/Narita/92A/98 8 (NS)
DQ064471 A/chicken/Guangxi/10/99 8 (NS)
DQ064472 A/chicken/Guangxi/9/99 8 (NS)
HQ117890 A/chicken/Guangxi/KMIII/1999 8 (NS)
EF620904 A/Korea/KBNP-0028/2000 8 (NS)
HQ871936 A/chicken/Korea/GH2/2007 8 (NS)
EU662955 A/chicken/Korea/S21/2004 8 (NS)
EU662954 A/chicken/Korea/S21/2004 8 (NS)
JX094858 A/chicken/Korea/01310/2001 8 (NS)
CY031279 A/duck/Hong Kong/702/1979-quail adapted 8 (NS)
CY031287 A/duck/Hong Kong/702/1979-quail adapted 8 (NS)
CY031271 A/duck/Hong Kong/702/1979-chicken adapted 8 (NS)
CY031263 A/duck/Hong Kong/702/1979 8 (NS)
CY005635 A/duck/Hong Kong/784/1979 8 (NS)
CY038422 A/chicken/Pakistan/UDL-02/2005 8 (NS)
CY038406 A/chicken/Pakistan/UDL-04/2006 8 (NS)
CY038414 A/chicken/Pakistan/UDL-01/2005 8 (NS)
CY038446 A/chicken/Pakistan/UDL-03/2005 8 (NS)
CY038454 A/chicken/Pakistan/UDL-03/2007 8 (NS)
CY073804 A/duck/Malaysia/2001 8 (NS)
AB473942 A/duck/Hokkaido/49/98 8 (NS)
AB262467 A/duck/Hokkaido/9/99 8 (NS)
KC162240 A/baikal teal/Xianghai/421/2011 8 (NS)
JN852807 A/chicken/Korea/KNUWSJ09/2009 8 (NS)
JN852799 A/chicken/Korea/KNUSWR09/2009 8 (NS)
JN852783 A/duck/Korea/KNUDPJ09/2009 8 (NS)
CY038430 A/chicken/Pakistan/UDL-01/2006 8 (NS)
FJ384747 A/chicken/Jiangsu/7/2002 8 (NS)
EU365375 A/chicken/Hubei/C1/2007 8 (NS)
FJ384755 A/chicken/Jiangsu/7/2002 8 (NS)
GU050283 A/avian/Saudi Arabia/910134/2006 8 (NS)
CY076727 A/chicken/Emirates/R66/2002 8 (NS)
Isolate1 S8
CY099349 A/chicken/India/IVRI-0004/2010 8 (NS)
CY038398 A/chicken/Pakistan/UDL-01/2007 8 (NS)
CY038470 A/chicken/Pakistan/UDL-02/2008 8 (NS)
CY038438 A/chicken/Pakistan/UDL-02/2006 8 (NS)
CY038462 A/chicken/Pakistan/UDL-01/2008 8 (NS)
CY038478 A/chicken/Pakistan/UDL-03/2008 8 (NS)
CY038486 A/chicken/Pakistan/UDL-04/2007 8 (NS)
JN540068 A/chicken/Sihala/NARC-12103/2008 8 (NS)
JN540076 A/chicken/Attock/NARC-14994/2009 8 (NS)
CY068647 A/chicken/Chandigarh/2048/2003 8 (NS)
CY068655 A/chicken/Orissa/2317/2004 8 (NS)
Isolate2 S8
CY068663 A/chicken/Haryana/2424/2004 8 (NS)
FJ605500 A/watercoot/Haryana/5844/2005 8 (NS)
CY068675 A/chicken/Gujarat/3697/2004 8 (NS)
GU064948 A/chicken/Tripura/105131/2008 8 (NS)
HM370053 A/chicken/Uchal/8286/2006 8 (NS)
HM370060 A/chicken/Uchal/8293/2006 8 (NS)
CY099365 A/chicken/India/IVRI-0011/2011 8 (NS)
GQ148850 A/chicken/Israel/182/2008 8 (NS)
GQ148849 A/chicken/Israel/54/2008 8 (NS)
JQ254968 A/chicken/Israel/1067/2010 8 (NS)
JQ254973 A/chicken/Israel/1167/2010 8 (NS)
JQ254978 A/chicken/Israel/1184/2010 8 (NS)
JQ254983 A/chicken/Israel/1293/2010 8 (NS)
JQ254958 A/chicken/Israel/32/2009 8 (NS)
GQ148851 A/chicken/Israel/184/2009 8 (NS)
GQ148861 A/chicken/Israel/953/2007 8 (NS)
GQ148859 A/chicken/Israel/883/2008 8 (NS)
GQ148858 A/chicken/Israel/869/2007 8 (NS)
GQ148860 A/chicken/Israel/951/2007 8 (NS)
GQ148853 A/chicken/Israel/330/2008 8 (NS)
GQ148855 A/turkey/Israel/689/2008 8 (NS)
GQ148857 A/chicken/Israel/702/2008 8 (NS)
GQ148848 A/chicken/Israel/1040/2007 8 (NS)
GQ148852 A/chicken/Israel/310/2008 8 (NS)
GQ148856 A/chicken/Israel/694/2008 8 (NS)
IL/90658/00 like
DQ683032 A/chicken/Israel/786/2001 8 (NS)
DQ683037 A/turkey/Israel/1209/2003 8 (NS)
DQ683033 A/turkey/Israel/810/2001 8 (NS)
DQ683036 A/turkey/Israel/1013/2002 8 (NS)
DQ683031 A/turkey/Israel/619/2002 8 (NS)
DQ683047 A/chicken/Israel/90658/2000 8 (NS)
EF492339 A/chicken/Israel/793/2005 8 (NS)
EF492331 A/chicken/Israel/909/2005 8 (NS)
EF492340 A/chicken/Israel/853/2005 8 (NS)
GQ148854 A/chicken/Israel/375/2007 8 (NS)
DQ683045 A/chicken/Israel/1953/2004 8 (NS)
EF492335 A/turkey/Israel/425/2005 8 (NS)
DQ683039 A/chicken/Israel/1376/2003 8 (NS)
DQ683041 A/chicken/Israel/1475/2003 8 (NS)
DQ683046 A/chicken/Israel/1966/2004 8 (NS)
DQ683044 A/chicken/Israel/1808/2004 8 (NS)
EF492338 A/turkey/Israel/747/2005 8 (NS)
EF492341 A/turkey/Israel/884/2005 8 (NS)
EF492334 A/chicken/Israel/282/2005 8 (NS)
DQ683026 A/turkey/Israel/89/2005 8 (NS)
EF492337 A/avian/Israel/584/2005 8 (NS)
EF492333 A/chicken/Israel/178/2006 8 (NS)
EF492336 A/chicken/Israel/554/2005 8 (NS)
CY087828 A/turkey/Wisconsin/1/1966 1966/06/11 8 (NS)
CY093092 A/quail/Lebanon/272/2010 8 (NS)
CY093100 A/quail/Lebanon/273/2010 8 (NS)
AB256686 A/chicken/Kobe/aq26/2001 8 (NS)
AB256718 A/chicken/Yokohama/aq134/2002 8 (NS)
KC693644 A/white-fronted goose/Korea/20-36/2007 8 (NS)
JX437687 A/egret/Hunan/1/2012 8 (NS)
KC209499 A/wild bird/Korea/8G39/2005 8 (NS)
0.05
